# Development of a smartphone enabled, paper-based quantitative diagnostic assay using the HueDx color correction system

**DOI:** 10.1371/journal.pone.0311343

**Published:** 2024-10-04

**Authors:** Nidhi Menon, David Beery, Prava Sharma, Adrian Crutchfield, Leah Kim, Aaron Lauer, Ayesha Azimuddin, Brianna Wronko-Stevens

**Affiliations:** HueDx, Inc., Philadelphia, Pennsylvania, United States of America; Old Dominion University, UNITED STATES OF AMERICA

## Abstract

Color correction is an important methodology where a digital image’s colors undergo a transformation to more accurately represent their appearance using a predefined set of illumination conditions. Colorimetric measurements in diagnostics are sensitive to very small changes in colors and therefore require consistent, reproducible illumination conditions to produce accurate results, making color correction a necessity. This paper presents an image color correction pipeline developed by HueDx, Inc., using transfer algorithms that improve upon existing methodologies and demonstrates real-world applications of this pipeline in colorimetric clinical chemistry using a smartphone enabled, paper-based total protein diagnostic assay. Our pipeline is able to compensate for a variety of illumination conditions to provide consistent imaging for quantitative colorimetric measurements using white-balancing, multivariate gaussian distributions and histogram regression via dynamic, non-linear interpolating lookup tables. We empirically demonstrate that each point in the color correction pipeline provides a theoretical basis for achieving consistent and precise color correction. To show this, we measure color difference with deltaE (ΔE00), alongside quantifying performance of the HueDx color correction system, including the phone hardware, color sticker manufacturing quality and software correction capabilities. The results show that the HueDx color correction system is capable of restoring images to near-imperceptible levels of difference independent of their original illumination conditions including brightness and color temperature. Comparisons drawn from the paper-based total protein assay calibrated and quantified with and without using the HueDx color correction pipeline show that the coefficient of variation in precision testing is almost twice as high without color-correcting. Limits of blank, detection and quantitation were also higher without color-correction. Overall, we were able to demonstrate the HueDx platform improves reading and outcome of the total protein diagnostic assay and is useful for the development of smartphone-based quantitative colorimetric diagnostic assays for point-of-care testing.

## Introduction

Color correction refers to any process that alters the perceived color of light. In the context of this paper, we refer to color correction specifically as the application of altering a digital image’s perceived color to bring it in alignment with its quantitative, measured color. Refining the color correction process is vital as images captured through analog or digital means rarely reflect the actual color of the captured scene. This is due to the standard illuminant, otherwise known as the source of light lighting the scene being captured. Observing different lighting sources reveals that soft candlelight imbues scenes with a warm, deep yellow glow, while a daylight LED light bulb can cause the glow to be pure white to cool blue. The international commission on illumination defines this as the color temperature where: “… a Planckian radiator whose radiation has the same chromaticity as that of a given stimulus” [1]. The source of light in a scene could cause our perception of white to range anywhere from red to blue. Due to this wide range of possible color temperatures and brightness, many techniques have been developed in digital image processing to correct for these effects. Software such as Adobe Photoshop, Darktable, GIMP and others contain many tools to adjust colors and light levels manually. In addition to the manual tools that are used to correct digital photos, many automated processes have also been developed to balance colors and prevent under or over-exposure. Modern smartphone camera applications such as those found on Apple iPhones and Android phones attempt to automatically correct the scene’s illumination temperature via a process called auto white balancing. There are also tools such as Google’s NightSight that allow the camera sensors to acquire more scene color information before producing a final image of a dimly lit scene. However, any industry, application or use case that requires consistent, measurable accuracy of colors needs a color correction process in place beyond what these products can supply.

In order to ensure color constancy, the most common approach is to use a predefined color set that is placed within the scene of an image being taken [2]. Examples of this approach are seen in the Pantone Color Match Card or the X-Rite ColorChecker. Because these devices contain a preset number of colors with known quantitative measures that cover a large range of the visual spectrum, the digital software can approximate, with great precision, the initial illumination conditions as well as what the scene would look like under different illumination conditions [3–5]. This allows a scene-invariant method for measuring colors and ensuring their constancy in design and print applications, as well as calibrating hardware to more accurately represent the perceived colors. In order to convert the scene and the color constancy device into the required illumination conditions, the applications responsible for processing the images apply color transfer methodologies. It is therefore possible to create a mathematical model that transforms the colors from the captured scene into the colors that are expected based on the device configuration and color set [2]. This same transform can be applied to the entire image and results in a final digital image that more closely represents the true colors of the scene, independent of the illumination conditions at the time. Several approaches have been developed to computationally model this process [3] with the more common approaches being histogram matching (HM) [6], Monge-Kantorovich Linearization (MKL) [7] and multivariate gaussian distributions (MVGD) [8].

The HueDx color correction system, comprised of the HueCard and HueTools iOS application, applies many of the main theories discussed above by treating the HueCard as a means of achieving color constancy through application of custom methods for color transfer. This is achieved through three primary steps. The first is a custom sticker design that utilizes a color gamut based upon the original ColorChecker design, but the selected colors have been improved to capture a broader range of possible transforms [9], The second is a software component that is capable of masking irrelevant context during the color transfer process by applying a pixel-wise boolean mask to the images. Instead of relying on a global transfer, HueTools is capable of automatically selecting only the known color regions and applying a local color transfer across the card to correct any colorimetric assays within the card’s region of interest. Third, state-of-the-art color transfer algorithms that have improved upon existing methodologies such as HM, MKL and MVGD as well as introducing new ones such as dynamic, non-linear interpolated lookup tables (LUTs) (DNIL).

**Fig 1:**
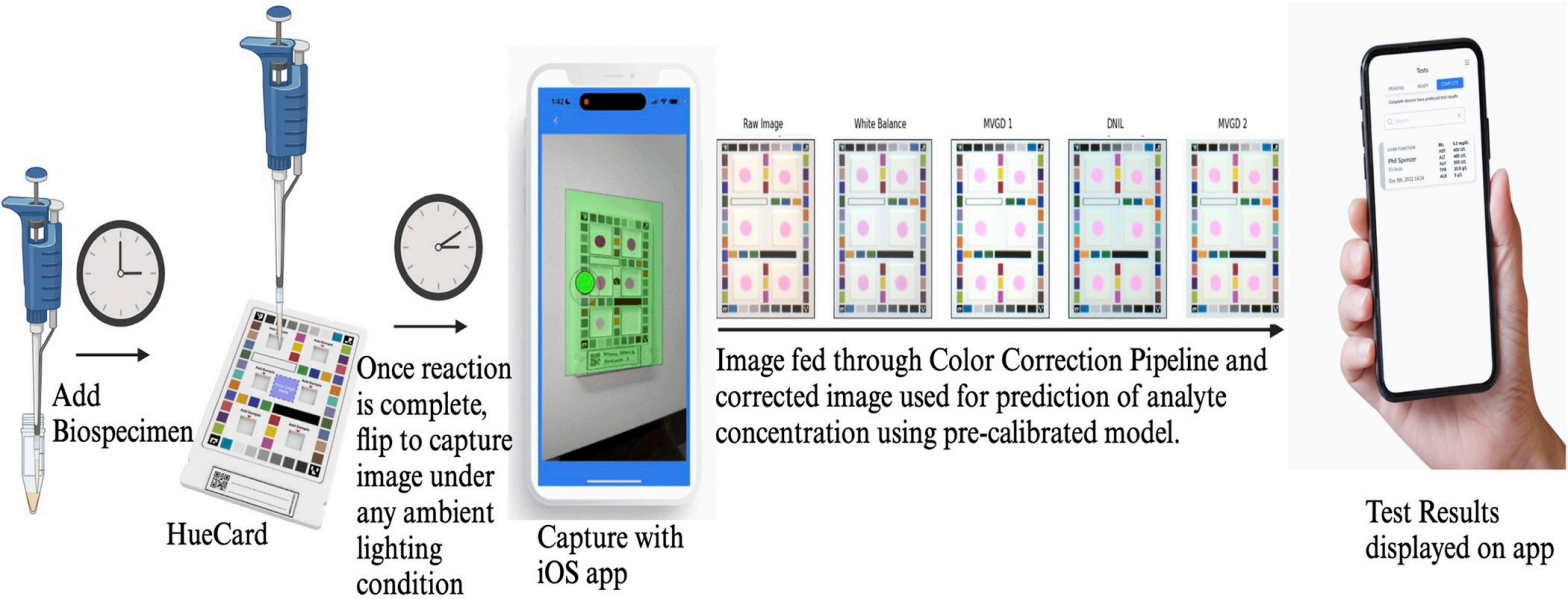
The HueDx color correction system and its application in colorimetric assays. The HueDx color correction system consists of the HueCard, HueTools iOS application and the backend color correction pipeline. The HueCard is lined with a custom color correction sticker in its perimeter and the wells in the card can be customized with various paper-based membranes for colorimetric assays. Once the biological specimen is added on the paper pad on the card, and the reaction is complete, an image of the HueCard is captured with the iOS application (supported by iPhones 11 and later), passed through our proprietary color correction pipeline, following which the analyte is quantified with high accuracy and precision using a pre-calibrated model for the specific analyte.

Together, these steps allow the HueDx system to be a compact and fully featured color correction platform that restores captured colorimetric assays to their expected colors under standard, laboratory illumination conditions. **Fig 1** depicts the flow of the system for its application in colorimetric assays. To demonstrate the effectiveness and accuracy of the system, we will be empirically measuring the physical and software limitations of the system at several potential failure points. These points include the camera phone sensor capturing the HueCard image, the custom color correction sticker and its consistency off the printer, the natural variation in real-world illuminations conditions, the final effectiveness of the color correction pipeline itself, and the real-world application of the color correction system using a total protein diagnostic assay.

## Methods

### Design and manufacturing of the HueCard and custom sticker

The HueCard was designed to incorporate a wide variety of paper-based materials. The HueCard was designed using SOLIDWORKS and is 3 inches wide and 5 inches in length, with a top and bottom that could be 3D printed (0.2inches thick) and assembled or injection molded with the desired material for analytical testing. The HueCard sticker was designed to incorporate an enhanced version of the original ColorChecker palette in which 24 colors were selected to reflect primary, secondary and tertiary colors as well as a grayscale spectrum and natural tones [10]. Two additional colors were added that extended the range of brightness from the darkest printable black to the brightest white representable on the material. These colors were arranged in patches that are mirrored across the x and y axes of the sticker design for a total of 48 color patches and 2 black/white references. By mirroring the colors across axes, it allows for the detection of local illumination problems such as shadowing or glare that may only affect a portion of the overall card or stickers. If the measured colors of oppositely placed color chips diverge, it indicates potential imaging problems due to local illumination which cannot be corrected through color correction alone. On each sticker, 6 holes were pre-cut to allow for biological samples to be placed into the card sample area and their colorimetric reactions to be measured on the opposite side of the card. The stickers were printed on an HP Latex 360 printer using ORACAL 651 as the printing medium. All colors were guaranteed a ΔEΔE00(ΔE2000 or ΔE00) score of <10 with all colors achieving a mean ΔEΔE00 of <3 and a max ΔEΔE00 score of 5.3. The ΔE00 metric was developed by the International Commission on Illumination (CIE) as a quantification of color difference: the distance or separation between two colors [11].

### Quantifying the phone sensor

The system currently supports several models of iPhone and the oldest model supported is the iPhone 11. The first step in validation of the HueDx system is to ensure that a phone camera sensor is capable of consistently reproducing the same color in any image it captures. To validate the phone sensor, two known colors were selected from the Pantone library: Cool Gray 1C and Neutral Black C. The whitest printed chip (Cool Gray 1C) was used as per the ASTM standard for verifying the performance of color measurement [12]. The blackest printed chip was used to verify that the extremes of reflectance did not influence the repeatability of the iPhone. These color chips were photographed with the iPhone 11, under identical lighting conditions, ten times each. The ΔE00 (ΔE00) standard was used to measure the difference between the captured colors.

A ΔE00 score of < = 1 is considered to have no perceptible difference between colors. A score of < = 3 may reveal slightly perceptible differences, but only under close examination. Scores < = 5 have small perceptible differences depending on factors such as color and brightness and is usually considered an industry standard for printing solid ink patches (ISO 12647–2). Finally, scores < = 8 tend to have noticeable differences that are visible from casual observation.

Each image that was taken had the Pantone color chip manually isolated from its surroundings, so the digital representation included only pixels that directly reflect the sensor’s measurement of the color chip. Each image was converted into the CIELAB color space, averaged and then ΔE00 is calculated pairwise between each of the images. The following metrics were reported: ΔE00 Max-This is the maximum ΔE measured between any two images that are furthest apart in color space and ΔE00 Mean- This is the average ΔE00 value across all pairwise comparisons of the images. The expectation is that a phone sensor should be able to consistently capture the same color with a ΔE00 Mean score of < = 1.0.

### Quantifying the quality of the stickers

Once the physical limits of the phone sensor were established, the quality of the stickers were validated. If the color patches on the stickers are consistently different, this will directly affect the system’s ability to correct colors in a precise and accurate way. To validate the stickers, 10 stickers were randomly selected from a batch of 400 printed stickers. These 10 stickers were photographed using the iPhone 11 under identical illumination conditions. Each sticker had all 24 color patch measurements and the white/black references compared pairwise with the other stickers. The ΔE00 Max and ΔE00 Mean were used to identify the maximum variation between stickers as well as the average variation between stickers. The expectation is that the ΔE00 Mean score should be < = 2.0 given the natural variation and physical limitations of printing discussed with our sticker manufacturer.

### Color correction pipeline

The captured images undergo a series of transformations through our proprietary color correction pipeline:

1. White balancing: This step utilizes a known value of white that is present on our sticker and then compares it to the image that was captured. This creates a 3x3 diagonal matrix for the red, green and blue color channels of the image. Multiplying this matrix with the image results in scaling the channel values up or down so that true neutral surfaces appear neutral in the image. This is a common process and was not enhanced or modified in any way.
2. MVGD 1: This second step utilizes a white balanced image as input and extracts all pixels from the colored patches on the sticker in the image (source) and then compares them to the expected color values of our sticker which was designed under the D50 illuminant assumption (target). The colors are represented in the CIELAB space. Our formula for the color transfer is the same as the original authors’ [8]. However, the application of the formula was modified to allow for color masking. This permits us to dynamically select only particular regions within the source and target when creating our color transfer matrix, rather than applying the process to the entire source and target images.
3. Histogram Regression via DNIL: Following the first MVGD color transfer, this step examines each individual color channel rather than all three simultaneously as in the MVGD. Each color channel is then shifted in such a way that the source distribution of colors more closely resembles the target distribution of colors. Each color channel of the source image has its cumulative distribution function calculated. *F*_1_() for the source image and *F*_2_() for the target image. For each value *V*_1_ in the source color channel, we find the target value *V*_2_ such that *F*_1_(*V*_1_) = *F*_2_(*V*_2_). This process is applied to each pixel of the source image The individual color channels are then recombined into a single image. Two aspects of this process were improved. The first is color masking, as with the MVGD step, we can apply this algorithm to only particular areas of the image and use just the color patches instead of the entire image. The second part is that we have added support for both linear and nonlinear interpolants in a dynamically selected method which we call DNIL. Given a set of possible interpolants A, we are able to optimize the histogram regression f() such that the interpolant x satisfies *max_x∈A_*f(x) thereby selecting the optimal histogram regression method. This dynamic method selection makes no assumptions about color distributions and optimizes results on a per-image basis. Traditional histogram matching uses a linear piecewise interpolant to extrapolate values in an 8-bit unsigned integer numerical space. The HueDx system’s approach supports more advanced interpolation in the more precise 32-bit float space so that color transformation results can utilize a wider range of values and become less prone to precision loss.
4. MVGD 2: After the previous three steps, a final application of MVGD, identical to the first is reapplied to the source image resulting in a color-corrected image that can be used for colorimetric analyses.

### Intra-site and inter-site consistency of the stickers

The final step to validating the system was to measure performance of the color correction pipeline itself. The first step was to capture “real-world” illumination variations and measure their ΔE00 differences without any correction. For this purpose, 5 locations were chosen to reflect a variety of illumination:

1. Noon/Vertical daylight ~5500K-5800K (Herein called 5800K)
2. Bright LED lighting at 5000K
3. Halogen lighting at 4000K
4. Incandescent lighting at 3000K
5. Dim lighting at 2700K

Each of these locations had 3 images captured with the iPhone 11. To measure the sticker’s captured color difference from a known color, the sticker manufacturer used an X-Rite i1Pro2 spectrophotometer to verify the stickers’ color profile. Across all 24 color patches printed on the stickers, a ΔE00 Mean score of 2.9 was achieved with a ΔE00 Max score of 5.3. The images were compared in two ways: intra-site consistency to ensure the site itself is stable and inter-site consistency to demonstrate the radical shift in color possible under different conditions and before correction. Both methods utilized the ΔE00 Max and Mean metric discussed earlier.

The color correction pipeline was then applied to the images and the sticker color patches re-measured to determine how far apart their ΔE00 measurements are from each other and from their ground truth color. The expectation is that the measured Mean and Max values will be >2.9 and >5.3 respectively. This hypothesis is motivated by the idea that natural variations in the printing process as well as real-world conditions are naturally bound at the lower level to the measured quantities from the manufacturer’s spectrophotometric QC process. Therefore, any achievable performance will be greater than the QC levels discussed above.

### HueTools application development for image capture

HueTools Mobile serves as a handheld companion for image capture. The mobile application was written in Apple’s native Swift programming language and utilizes AVFoundation, CoreMotion and Vision frameworks/libraries to allow (near) full control of device and camera sensor functionality. This control allows for the mobile application to handle custom features including, but not limited to, point of focus, torch (flash) intensity, pixel density, image resolution, capture angle, etc. All captured images are processed and streamed in a raw and uncompressed format to assure max pixel density and color clarity. Image data is then sent via a secure API to the backend services for AI/ML processing though the applicable pipelines.

### Total protein diagnostic assay calibration and quantification

A cellulose-based paper pad (CFP-DBS, I.W Tremont) was used for the assay and embedded into the HueCard. The assay uses a Total Protein Reagent (Sigma, T1949). A known concentration (NIST Traceable) of total protein (Level F, Verichem, 9460) is diluted with 1X PBS to create the standard concentrations ranging from 0-10g/dL. One standard concentration is run per card (n = 6). 100 uL of the reagent are added to the paper, dried for 10 min, followed by 10uL of the sample. The amount of total protein is quantified after 10 min of incubation using the HueTools image capture software. Images are captured in 3 different locations, with iPhone 13 and iPhone 14. Images are either passed through the HueDx color correction software or left as raw images. The HueCard was segmented from the image, the ROIs of each individual sample well were isolated. These images were used to train an EfficientNetv2-B0 convolutional neural network which was built with a single-neuron dense layer using RELU activation for predicting regression values [13]. All model training and evaluation was performed in Tensorflow. The training data was comprised of input-output pairs {*x_i_, y_i_*} where x was the isolated sample ROI scaled to a size of 224x224 pixels, and y is the ground truth concentration of total protein in the sample. Model training and validation was performed using an 80–20 dataset split. The best performing model was saved for evaluation [14]. At inference time, all new ROIs from the test set were scaled to 224x224 pixels and processed through the convolutional neural network to produce regression values. To show the difference between quantifying total protein levels, the precision, linearity, and limits of detection were calculated with and without the images being processed through the color correction pipeline. Precision is measured with calibrator samples in a pseudo matrix, with concentrations 1.5 g/dL, 4.0 g/dL, 6.5 g/dL, 7.5 g/dL and 9 g/dL. Images were captured in 3 unique sites with different lighting conditions, with 3 different users and 3 different iPhones (11, 13, and 14). Data for precision was analyzed using the Analyse-it plug-in on Excel, following guidelines in EP05-A2 by the Clinical Laboratory Standards Institute(CLSI) [15]. To simulate CLSI guidelines in our limits of detection study, we tested blank (0 mg/dL) devices and concentrations close to the predicted limit of detection (LoD), which were 1.6, 1.8,2.0 and 2.2 g/dL of total protein. The limit of blank (LoB) and LoD were calculated following guidelines from CLSI EP-17(A2) using the Analyse-it Plugin [16]. The limit of quantitation (LoQ) was calculated based on the precision profile CV at 20%. Images for each card (n = 6) of the concentrations were taken in 3 separate locations with varied lighting conditions, and the LoB, LoD and LoQ were calculated with and without color correction. Linearity of the predictive model with and without color correction was also calculated. The analyse-it plug-in for excel was used to perform the data analysis, following the guidelines in CLSI EP06-A2 [17]. To test the applicability of the model in a point-of-care setting, 5 pooled samples of serum(2.2 g/dL,2.4 g/dL,3.7g/dL,4.8g/dL, and 7.0g/dL) as well as 2 pooled samples of plasma (3.0 g/dL, 6.0 g/dL) were both tested on the platform and compared with the reading on the Roche cobas c311.

## Results

### Sticker design and HueCard

**Fig 2:**
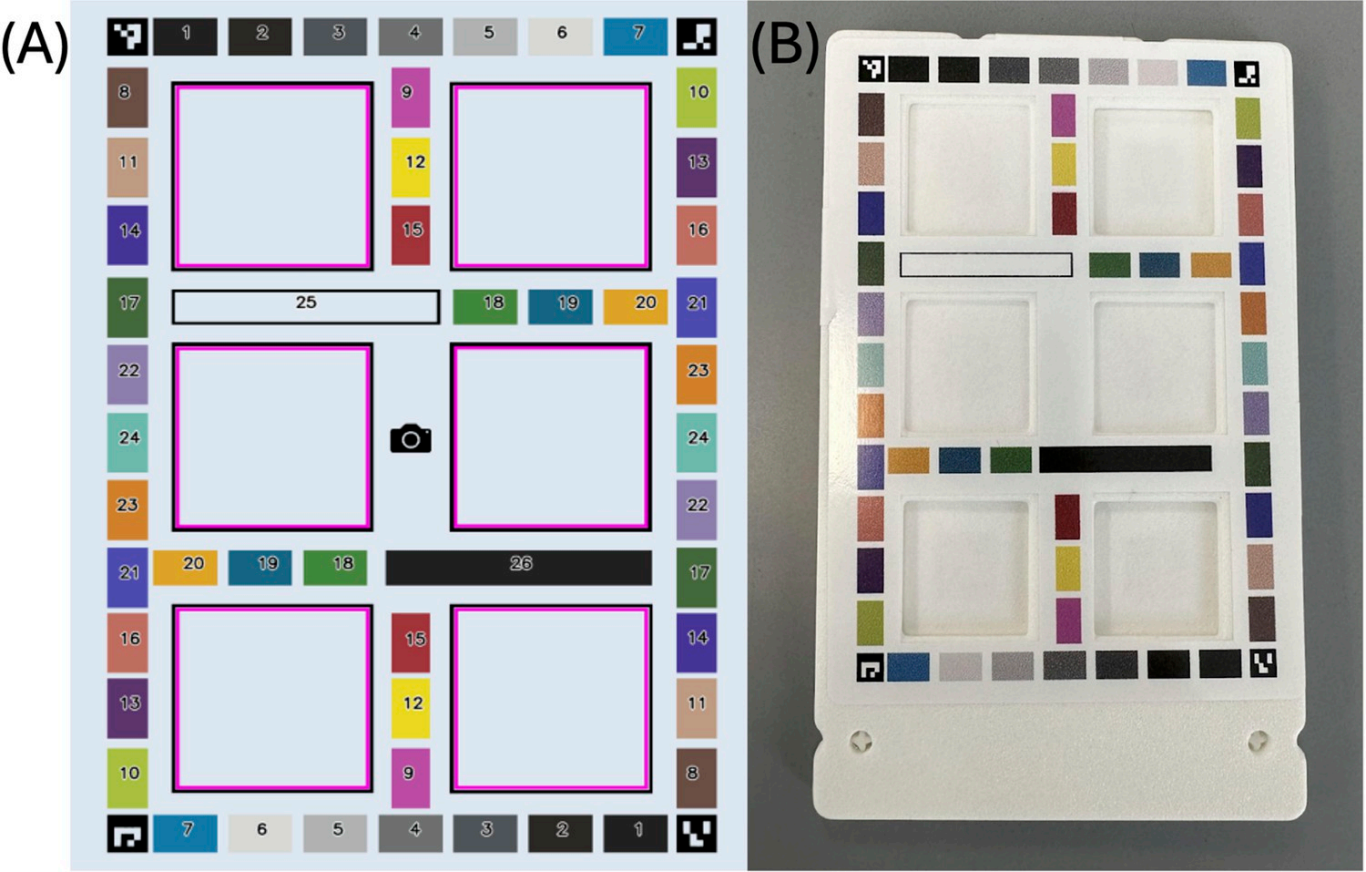
Sticker design and HueCard. **(A)** The sticker schematic with the individual color chips numbered for reference. **(B)** The sticker on the physical 3D printed HueCard used for the experiments in the study.

**Fig 2(A)** shows the sticker design along with the numbered patches used for the analysis of the following studies. **Fig 2(B)** shows the sticker applied to the HueCard. The HueCard was customized to contain pantone chips for the phone sensor quantification studies.

### Phone sensor quantification

**Fig 3:**
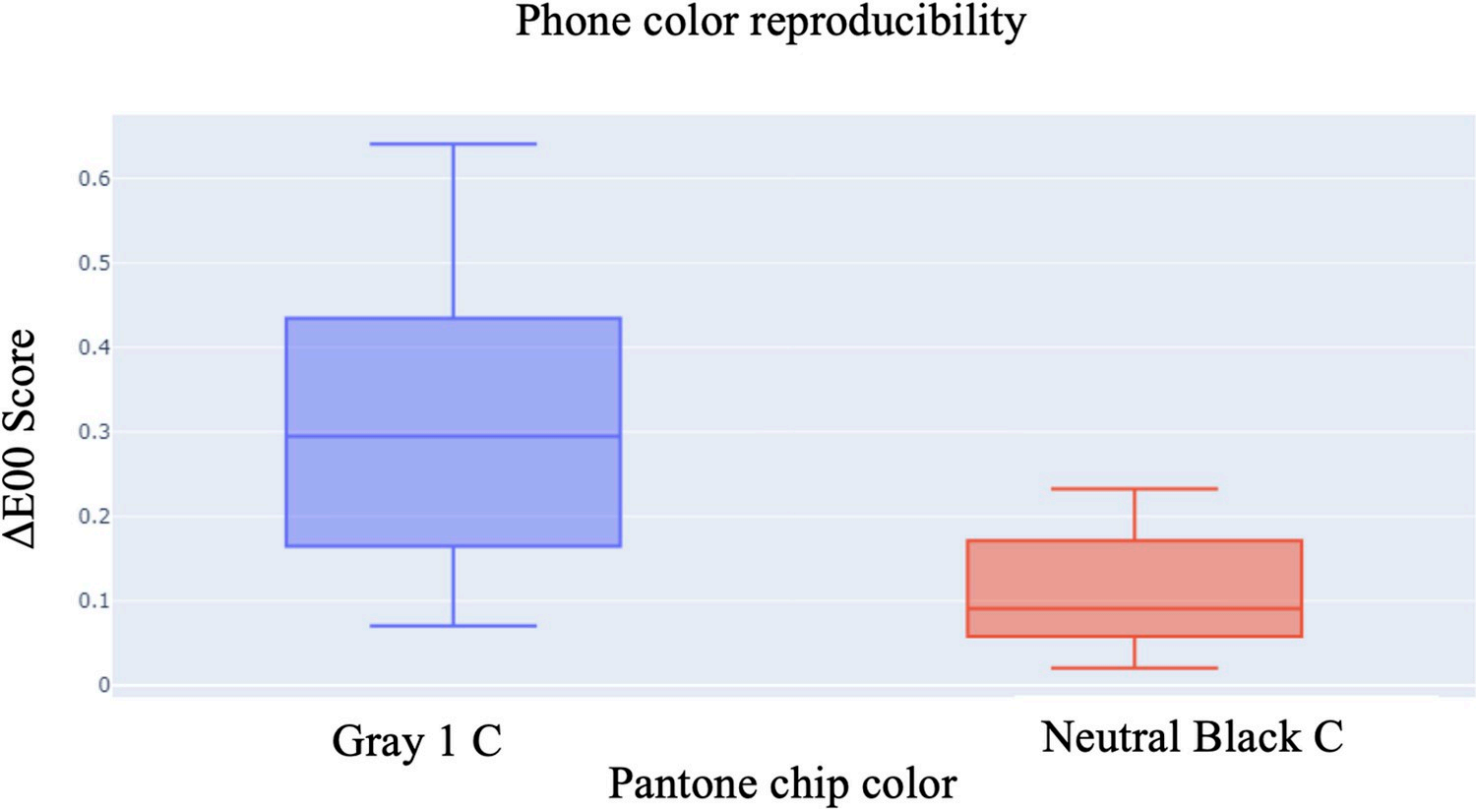
Phone sensor quantification indicates acceptable color reproducibility. Distribution of the measurements for each pantone chip color measured. The box plots show the median and range of the measurements. The whiskers represent the maximum and minimum values (excluding outliers). Mean ΔE00 score of Cool Gray 1C was 0.310.16 and the ΔE00Max was 0.64. Mean ΔE00 score of Neutral Black C was 0.110.06 and the ΔE00Max was 0.23.

10 images were taken of the Cool Gray 1C and Neutral Black C color chips each for a total of 20 images. The chip was isolated from the surrounding image which produced a solid color image for analysis. The ΔE00 metric was applied pairwise for each color resulting in 45 intra-color measurements for each chip. **Fig 3** shows the color reproducibility is within the expected limits. Images used for sensor quantification are provided in **S2 File**.

### Sticker consistency quantification

**Fig 4:**
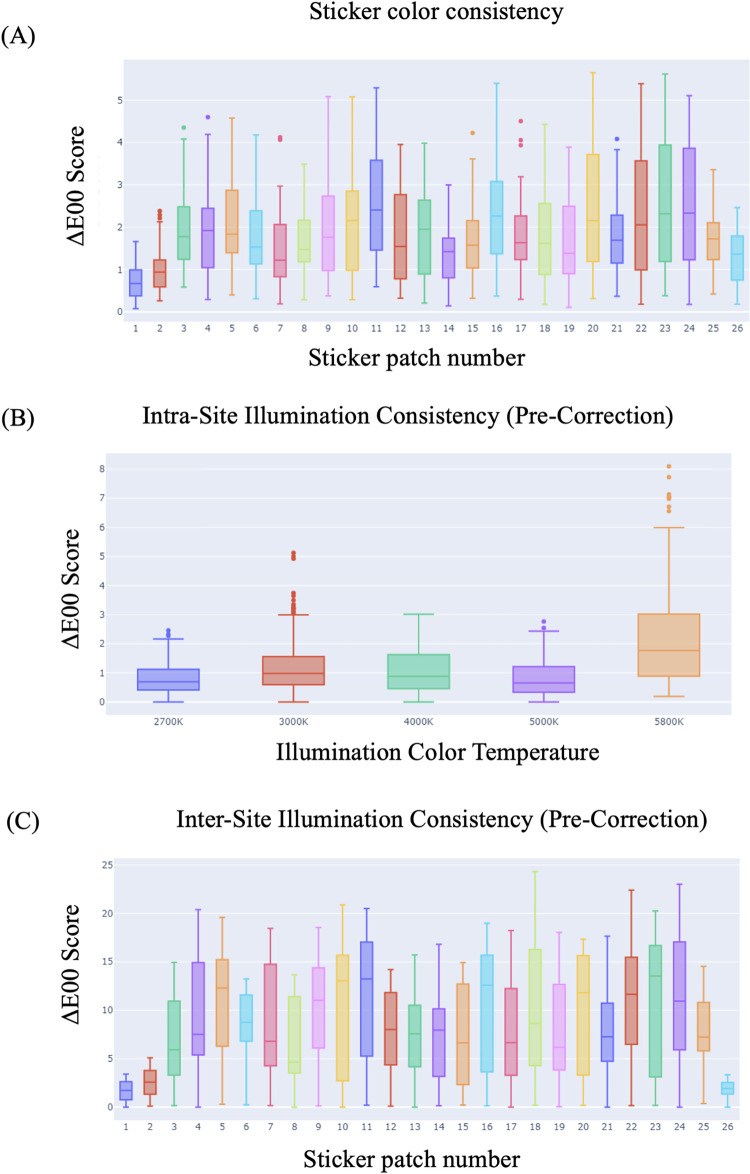
Characterization of the sticker patches. **(A).** Distribution of the ΔE00E scores for each of the individual sticker patches in the same lighting condition. The box plots show the median, range and outliers of the distribution. **(B)** Box plot showing the distribution (median, range and outliers) of ΔE00 values within a given site. **(C)** The ΔE00 score distribution (median and range) per sticker patch with inter-site illumination pre-correction. The whiskers represent the maximum and minimum values (excluding outliers).

The 10 sticker images were all compared pairwise for a total of 45 comparison combinations. Across all 26 color patches, this resulted in 1170 ΔE00 measurements. **Fig 4(A)** shows each color patch’s ΔE00 score compared to the other 9 images in the group. All median values for each patch fall below 2.5 ΔE units and the mean ΔE00 score is 1.84. All patches have similar consistency levels with an average standard deviation of 1 unit. These results indicate high degrees of consistency between printed stickers, independent of any correction process. ΔE00 data for the individual patches is available in **Table 1 in S1 File.** Images used for sticker consistency quantification are provided in **S3 File**.

### Real-world simulation: Intra-site and inter-site consistency of the stickers

The same color correction sticker was photographed 3 times at 5 distinct locations based on their illumination color temperature. Cards at each site had all color patches compared with each other and averaged for the intra-site ΔE00 metrics and then they were compared to their ground truth colors for an uncorrected ΔE00 measurement. **Fig 4(B)** shows the boxplot distribution of ΔE values at each location, organized by color temperature. The sites from 2700K-5000K show very tight distributions with their median values falling below 1. The 5800K site shows a larger spread of ΔE values but maintains a median and mean value under or close to 2. Overall, the intra-site agreement on average is 1.19 which strongly indicates an ability to capture consistent images in the same conditions, a reinforcement of the findings from the sticker consistency measures shown above.

**Table 2 in S1 File** looks at each site and measures the image’s ΔE distance from the ground truth color patches. As expected, the illumination color temperature drastically affects the appearance of the colors when captured digitally. The average ΔE00 score when comparing the images to the ground truth rendered color is 12.72. At this level, the color difference is easily and immediately noticed between images.

Comparing between sites, **Fig 4(C)** shows a boxplot of pre-corrected color agreement. As expected, drastic color differences are apparent between locations with 50% of the color patches exceeding a ΔE00 score of 8. **Table 3 in S1 File** shows the ΔE00 Mean score across all patches is 7.9 indicating easily visible differences between locations. Only the black or very dark patches (1, 2 and 26) show ΔE00 levels that indicate indiscernible color variations.

**Fig 5:**
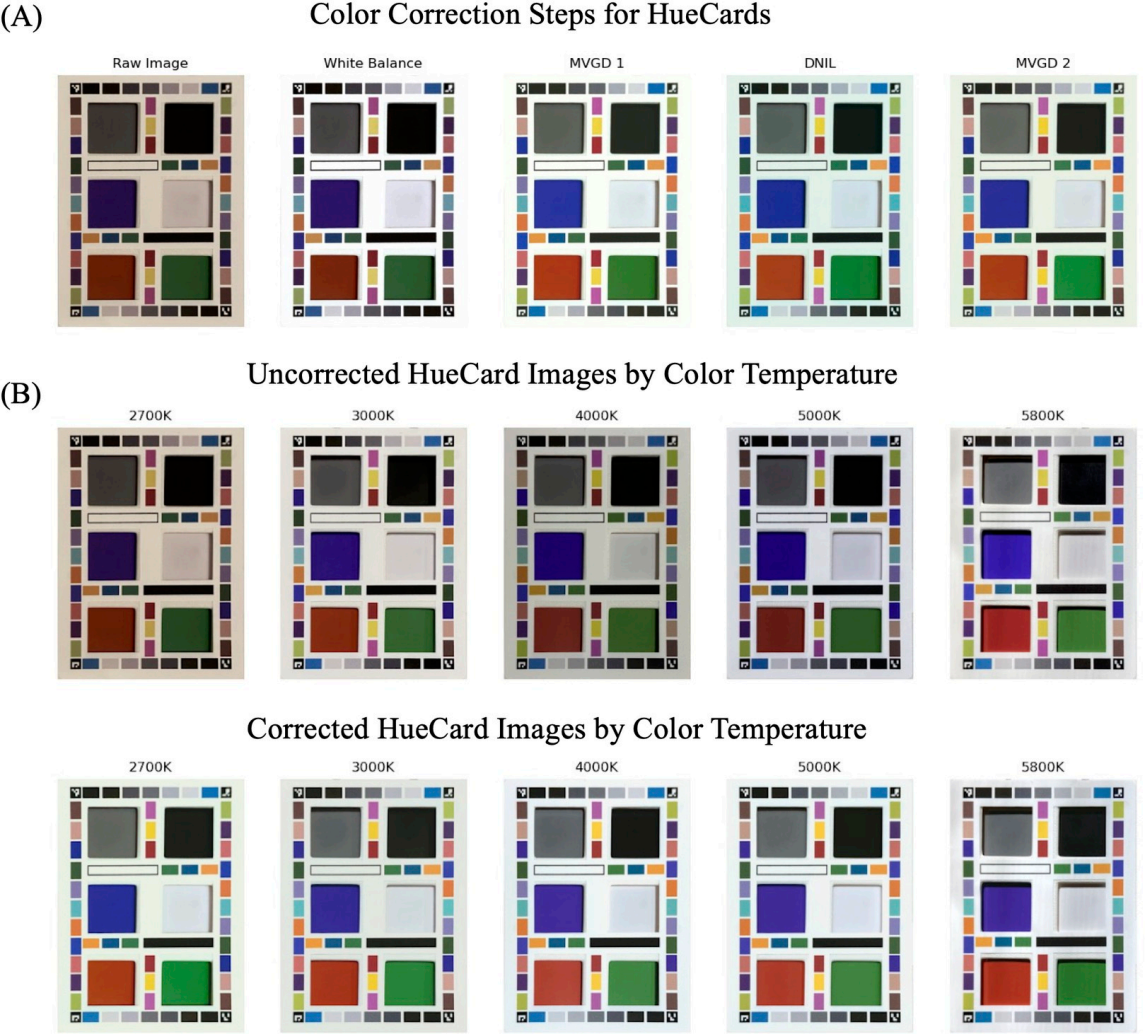
Color correction pipeline and application. **(A)** Series of images showing an example of the progression of the raw image through the color correction pipeline for a HueCard image captured under a 2700K color temperature. **(B)** HueCard images under various illumination conditions before and after the application of the HueDx color-correction pipeline.

While data from a single site demonstrates consistent, repeatable measurements between images (intra-site mean), the actual color varies greatly from the true color under the original illumination conditions. Our color correction pipeline (**Fig 5(A)**) was applied to the images and then the same metrics calculated.

**Fig 5(B)** shows the HueCards prior to correction and their appearance immediately post-correction. The perceptual difference in cards can be seen especially clearly at the 2700K-4000K level. Pantone color chips were also added into the HueCard to visually highlight the changes at various color wavelengths and clearly show the improvement of the digital color rendering after color correction.

**Fig 6:**
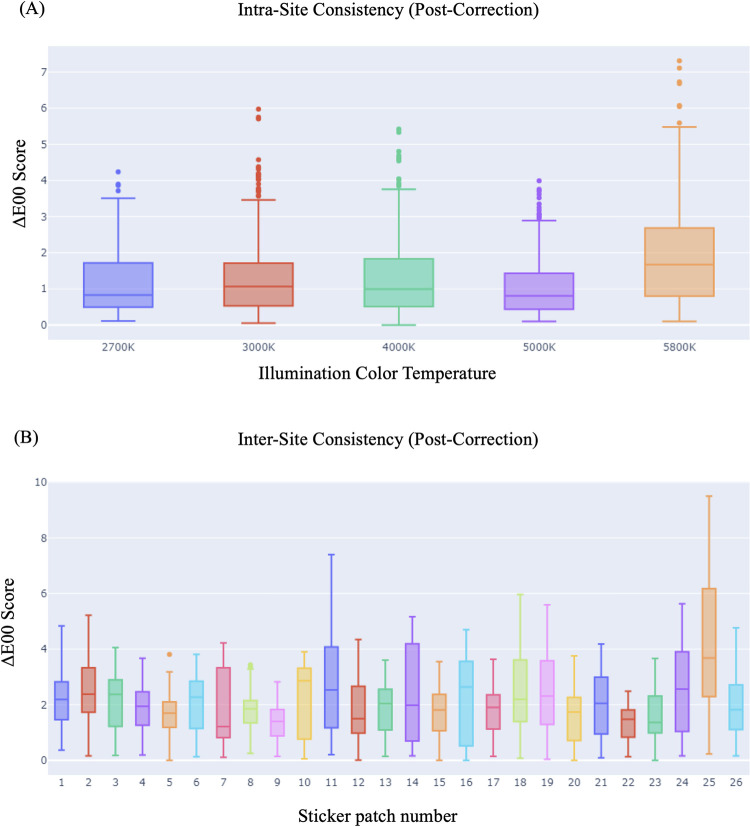
ΔE00 scores post correction for intra and inter-site show consistency in the pipeline’s ability restore illumination conditions to ground truth. **(A).** Distribution of the ΔE00 scores (median, range and outliers) of each of the color temperature post-correction. The whiskers represent the maximum and minimum values (excluding outliers) **(B)** The ΔE00 score distribution (median and range) per sticker patch with inter-site illumination post-correction. The whiskers represent the maximum and minimum values (excluding outliers).

**Fig 6(A)** re-measured the intra-site agreement levels after correction and indicates very little shift between images. This lack of change indicates that images taken in the same context are color corrected in a consistent way that does not artificially introduce new errors or problems. The Intra-Site measurements (**Table 1**) quantify this fact where a small 12.6% increase from 1.19 to 1.34 was seen when comparing intra-site images pre- and post-correction. This shows the scope of improvement after color correction when comparing the color patches to their ground truth values. The ΔE00 mean score pre-correction of 12.72 decreased 70% to 3.81 with only the 2700K location having a mean value above 4.0. **Fig 6(B)** shows the ΔE00 scores of each color patch when compared between locations. These scores (**Table 4 in S1 File**) directly reflect the HueCard system’s ability to bring images under varying illumination conditions back to a central illumination ground truth. The mean ΔE00 score across all color patches was 2.07, a 74% decrease from the pre-correction value of 7.9. Additionally, the average worst-case ΔE00 distance decreased from 16.48 to 4.53, a 73% drop. These numbers show that the correction process is precise and that the HueCards occupy a very tightly grouped color space together.

**Table 1:** Illumination temperature and ΔE00 scores after color-correction is applied for intra-site measurements.

Illumination Temperature	Intra-Site *Δ*E00 Mean	*Δ*E00 Mean from Ground Truth	*Δ*E00 Max from Ground Truth	Standard Deviation
~5800K	1.93	3.07	8.34	1.60
5000K	1.02	3.82	12.00	2.34
4000K	1.29	3.51	10.65	2.08
3000K	1.32	3.89	14.22	2.88
2700K	1.16	4.79	17.53	3.52
**MEAN:**	**1.34** (↑12%)	**3.81** (↓70%)	**12.55** (↓36%)	**2.63** (↓59%)

### Total protein diagnostic assay calibration and quantification

**Fig 7:**
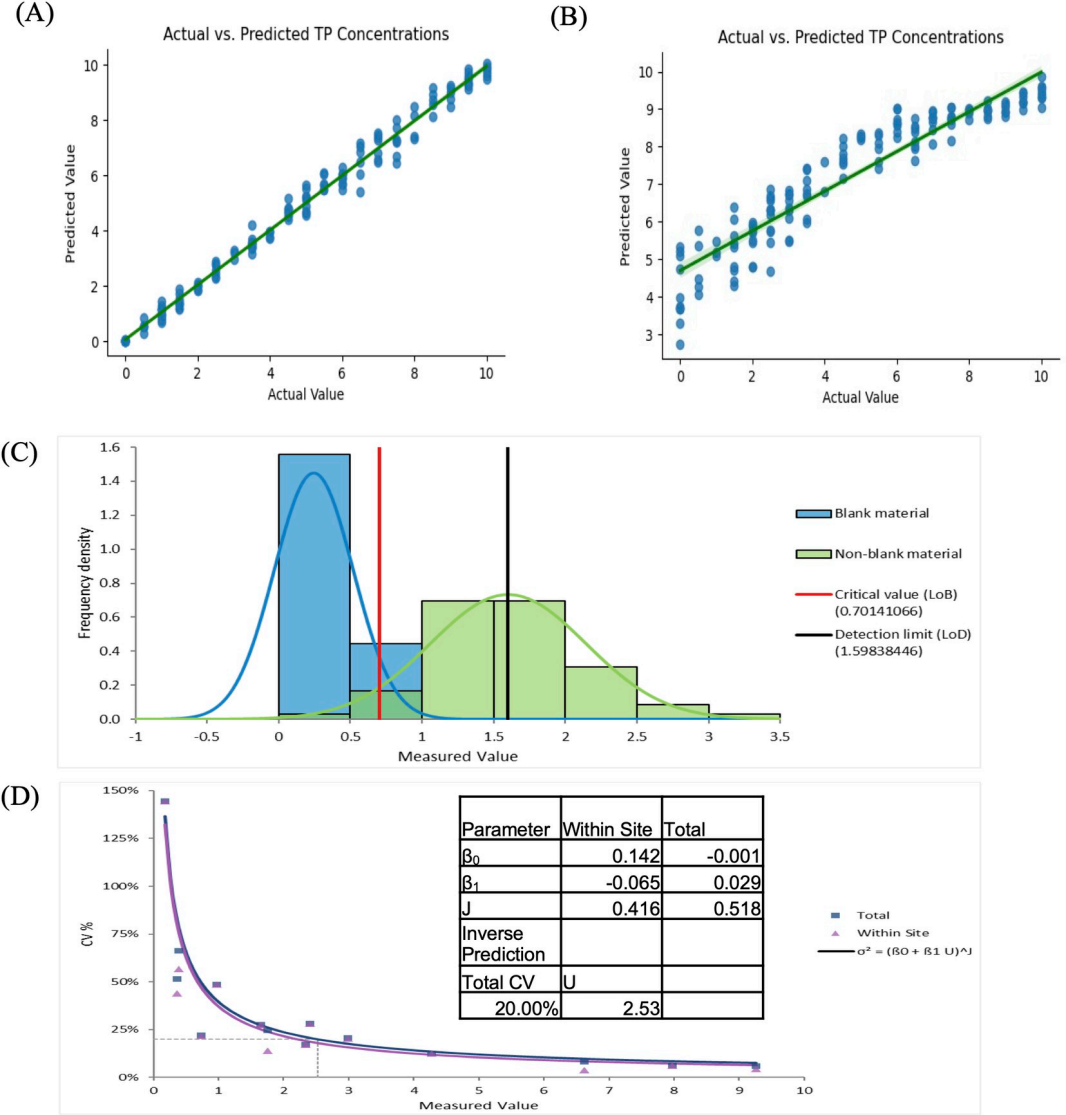
Calibration of the system and limits of detection. (A) Predicted concentrations using the model used post color-correction. (B) Predicted concentrations using the model used without color-correction. (C) The limits of blank and detection calculated using the normal quantile distribute of the predictions with known values of samples. (D) The %CV precision profile and the variance function describing the profile. The limit of quantitation determined as 2.53 at %CV = 20.

Calibration of the total protein assay resulted in color-corrected and non-color corrected prediction models that were used in subsequent studies. **Fig 7(A)** and **7(B)** show the performance of both the color-corrected and uncorrected images respectively for the range of concentrations that were tested. The color-corrected model consists of a linear relationship between the known and predicted concentrations, with r^2^ = 0.995, slope of 1.004, y-intercept of -0.012, and standard error of 0.0085. The model developed from uncorrected images had an r^2^ = 0.94, slope of 1.67, y-intercept of -7.27 and standard error of 0.0494 The color-corrected and uncorrected models were compared using ANOVA which showed significant difference between the two models (p < 1e-10), strongly preferring the color-corrected model. **Fig 1 in S1 File** shows the images of the cards with and without color correction for three unique total protein concentrations.

The precision of the models was assessed both with and without color-correction using the prediction models generated for each set using the calibration images. **Tables 2** and **3** show the predicted mean, standard deviation, and coefficient of variation (CV) for both models. The CV is consistently higher without the applied color-correction for both overall repeatability as well as reproducibility across the different sites.

**Table 2:** Total protein precision estimates for color-corrected HueCards.

Sample	Mean (g/dL)	Repeatability	Repeatability	Reproducibility	Reproducibility
		SD	% CV	SD	% CV
1.5 g/dL	1.820	0.344	18.90%	0.424	23.30%
4 g/dL	4.640	0.748	16.10%	0.939	20.20%
6.5 g/dL	6.959	0.593	8.50%	0.875	12.60%
7.5 g/dL	7.996	0.461	5.80%	0.461	5.80%
9 g/dL	9.374	0.380	4.10%	0.495	5.30%

**Table 3:** Total protein precision estimates for uncorrected HueCards.

Sample	Mean (g/dL)	Repeatability	Repeatability	Reproducibility	Reproducibility
		SD	% CV	SD	% CV
1.5 g/dL	1.355	0.332	24.5%	0.661	48.80%
4 g/dL	3.428	0.719	21.0%	1.312	38.30%
6.5 g/dL	5.834	0.713	12.20%	1.496	25.60%
7.5 g/dL	6.529	0.639	9.80%	1.725	26.40%
9 g/dL	7.984	0.313	3.90%	1.291	16.20%

The goal of the limits of detection study was to determine if the combination of a colorimetric assay, phone camera and software can demonstrate functional sensitivity for a point-of-care test for total protein. LoB is defined as the highest quantity value that could be reported for a blank material. LoD is defined as the minimum detectable value wherein the probability of a false negative is less than beta (5%), given the probability alpha (5%) of falsely claiming its presence. The LoB and LoD were calculated as 0.70 g/dL and 1.6g/dL respectively (**Fig 7(C)**). The prediction profile for CV was plotted and a variance function was fitted to calculate the LoQ where CV was equal to 20% (**Fig 7(D)**). LoQ was calculated to be 2.53 g/dL. When there was no color correction applied, LoB was calculated to be 0.52 g/dL and LoD was 1.72 g/dL (**Fig 2(A) in S1 File**). The prediction profile for CV and the variance function was unable to predict LoQ due to high %CVs across the range of concentrations (**Fig 2(B) in S1 File**).

**Fig 8:**
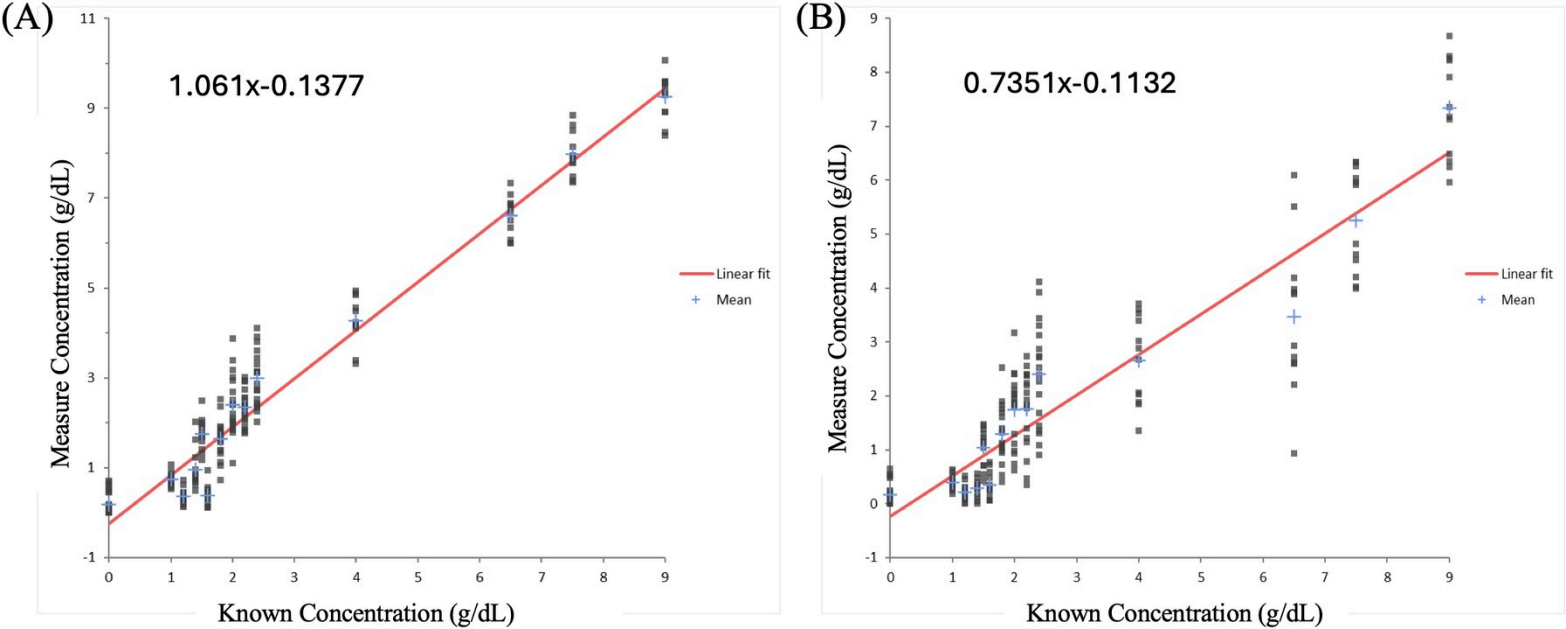
Assessment of linearity of both corrected and uncorrected models. **(A)** A least squares regression method for linear fit of measured values with known levels of total protein(g/dL) after color-correction. **(B)** A least squares regression method for linear fit of measured values with known levels of total protein(g/dL) without color-correction.

Linearity of the system was calculated using the limits of detection and precision dataset. The model predictions from corrected images produced a linear fit (**Fig 8(A)**), and no 2^nd^ or 3^rd^ order polynomial fit is statistically better than a linear fit at the 5% significance level. **Table 5(A) in S1 File** shows the parameters of the linear fit model compared to 2^nd^ and 3^rd^ order polynomials. For the images processed without correction through the uncorrected calibration model showed that both 2^nd^ and 3^rd^ order polynomials had parameters that were non-zero at the 5% significance level **(Table 5(B) in S1 File)**. **Fig 8(B)** shows the linear fit for the uncorrected model.

**Fig 9:**
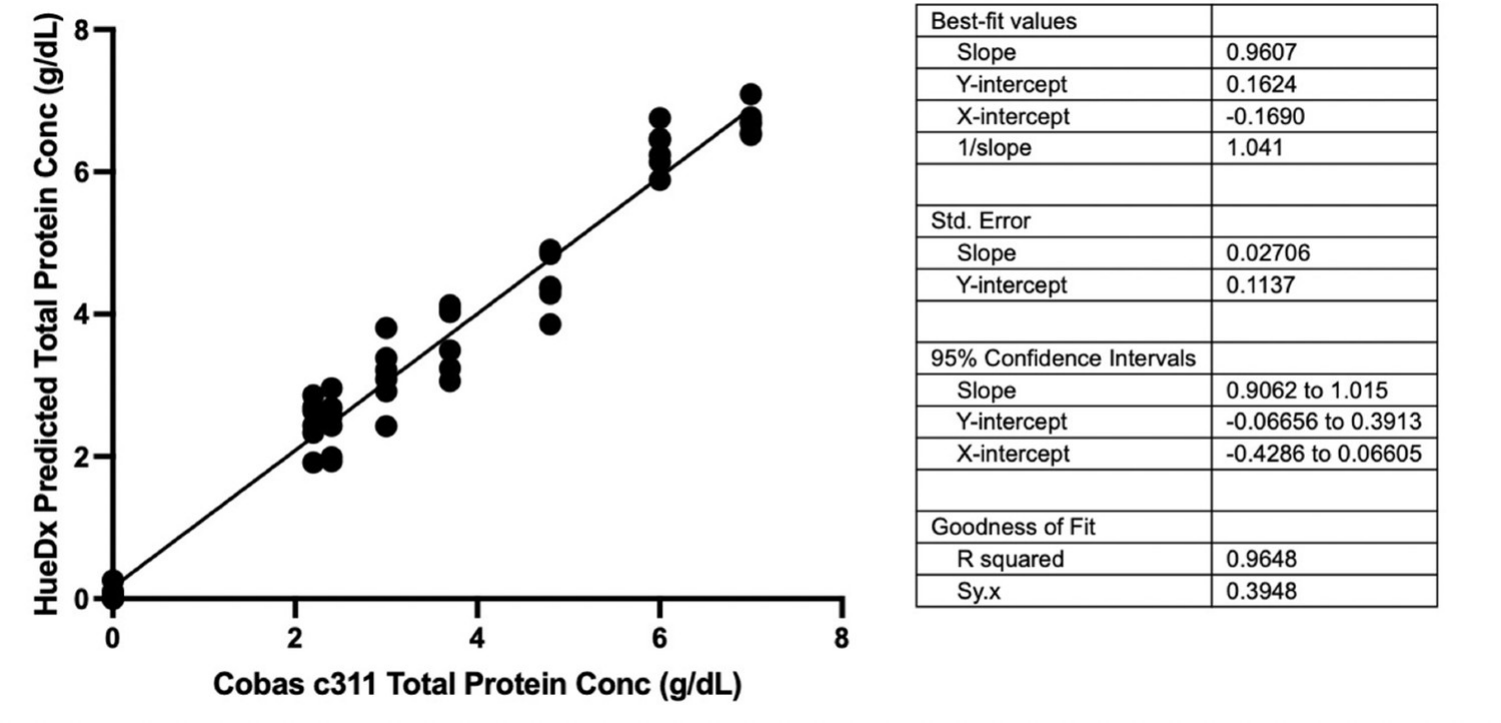
Comparison of the predictions of total protein in pooled plasma and serum samples from the HueDx system and the reference value from the Roche cobas c311.

**Fig 9** shows the results from the method of comparison for total protein, for which serum and plasma samples were measured in both Roche cobas c311 and on the HueDx system. Results from regression analysis demonstrated a linear relationship with a slope of 0.96 and intercept of 0.16.

## Discussion

In this paper, we have described an end-to-end color correction pipeline and quantified its performance within a range of real-world illumination conditions. We demonstrated that the use of an iPhone to digitally capture images of the HueCard device is precise and accurate to levels <1.0 ΔE00. Further characterization of the HueCard stickers shows that they maintain an average printing consistency of <2.0 ΔE00. In the context of varying illumination conditions, the ΔE00 Mean of the 26 sticker color patches from the ground truth colors was 12.72. After correction, the ΔE00 Mean of the sticker patches drops to 3.81. This level of corrective ability falls within our expectations given the previously discussed theoretical lower limit of 2.9 established in the intra-site consistency section. The most important quantity of this paper is the ΔE00 Mean between cards from the varying illumination conditions. Without any correction the sticker patches measure a ΔE00 Mean score of 7.9. Following the correction pipeline, the same images record a score of 2.07 which once again meets our expectations. Post correction, it is important to note that the ΔE00 Mean, Max and standard deviation scores are 2.07, 4.53 and 1.18. These numbers closely approach the theoretical sticker consistency limits elaborated in part 2 of the results section: 1.84, 4.22 and 1.00. This pattern lends further evidence to our system being able to correct for color differences to the theoretical limits imposed by the printer and physical materials. At this level of performance, the system is capable of correcting identical colorimetric results to a level of similarity that makes differences almost indiscernible.

An important factor to analyze is the intra-site ΔE00 mean after correction. To produce consistent results from identical illumination conditions, we must ensure that the intra-site ΔE00 score did not significantly change due to the corrective action taken on the images’ colors. Prior to correction the intra-site ΔE00 Mean score was 1.19. After correction the score was computed to be 1.34. Both scores are well below a threshold of 2 and indicate that our correction pipeline creates no discernible difference in the quality or precision of images taken in the same illumination context. During collection of the data across the illumination conditions, a possible relationship was noted between the strength of the light source as well as its color temperature (**Table 6 in S1 File**). The greatest outlier in the dataset occurs at a color temperature of 2700k and a lux value of 26. All the other sites had lux values close to or above 100 which may indicate that the strength of illumination at the time of photo capture may play a large role in the ability to adequately correct the HueCard back to its intended illumination target. Additional studies will be required to assess the correlation and draw conclusive results.

Prior to this experiment, the HueCard color correction pipeline was enhanced to operate in 32-bit color space without linear assumptions. An ablation experiment was run to determine the overall effect of this change and its contributions to more precise and accurate color correction. All the data and processes from this paper were run in an identical fashion, but the color correction pipeline was restricted to only using 8-bit color space representations and default linear assumptions. The results showed an average change of +4% across the final color-corrected ΔE00 scores. This confirms that removing linearity assumptions and increasing numerical precision results in more accurate color correction. **Fig 3 in S1 File** further shows the contributions of individual pipeline steps and the motivation for chaining particular color correction operations in the order selected for this paper.

Experimental results from the paper-based total protein diagnostic assay demonstrated the need for a color-correction pipeline in a colorimetric point-of-care, quantitative test. The color-correction pipeline was able to significantly improve the precision, linearity, and limits of detection of the assay. The poor performance of the diagnostic assay parameters without the application of the color-correction pipeline were evident in the results. The total protein assay serves as a proof-of-concept to demonstrate the effectiveness of the HueDx system. Close concentrations of total protein, 0.5g/dL or further apart, were well distinguished by the system and are a good indication of the performance of the system. For example, biological samples with predicate concentrations of 2.4 g/dL, 3.0g/dL, 3.7 g/dL, and 4.8 g/dL had mean (±SEM) HueDx predictions (n = 6) of 2.43 ±0.16g/dL, 3.14 ±0.19 g/dL and 3.67 ± 0.19 g/dL, and 4.44 ± 0.16g/dL respectively. The data supports the development of paper-based microfluidic testing for various analytes through colorimetric chemistry for applications in quantitative screening and companion diagnostics. Smartphone-based POC tests have been developed and reported in abundance in literature, however very few are able to translate to real-world applications [18–20]. Color correction methods to counter ambient lighting are critical in situations such as these wherein the measured outcome of a diagnostic test directly impacts health treatment plans. The perception of color varies based on the multiple factors affecting the ability of the intrinsic phone camera sensors to provide an accurate measure [21, 22]. The HueDx system has significant potential for providing accurate and precise color information using a simple mobile application, the color-correction pipeline and a quality-controlled color sticker. The ease of use of the platform makes it convenient to be used by anyone, anytime and anywhere.

## Supporting information

S1 File Supplemental figures and tables. (PDF)

S2 File Images used for phone sensor quantification. (ZIP)

S3 File Images used for sticker consistency quantification. (ZIP)

## References

[1] International Commssion on Illumination, International Commssion On Illumination C. CIE TN 013:2022 Terms related to Planckian radiation temperature for light sources. International Commssion on Illumination; 2022 Feb. doi: 10.25039/TN.013.2022

[2] Sunoj S, Igathinathane C, Saliendra N, Hendrickson J, Archer D. Color calibration of digital images for agriculture and other applications. ISPRS Journal of Photogrammetry and Remote Sensing. 2018;146: 221–234. doi: 10.1016/j.isprsjprs.2018.09.015

[3] Liu S. An Overview of Color Transfer and Style Transfer for Images and Videos. arXiv; 2022. doi: 10.48550/ARXIV.2204.13339

[4] Barbero-Álvarez MA, Menéndez JM, Rodrigo JA, Ramírez-Barat B, Cano E. Assessment of the Robustness of a Color Monitoring Chart Calibration Method for Crowdsourcing-Based Preventive Conservation. Applied Sciences. 2021;11: 10067. doi: 10.3390/app112110067

[5] Tominaga S, Schettini R, Trémeau A, Horiuchi T, editors. Computational Color Imaging: 7th International Workshop, CCIW 2019, Chiba, Japan, March 27–29, 2019, Proceedings. Cham: Springer International Publishing; 2019. doi: 10.1007/978-3-030-13940-7

[6] Gonzalez C R, Woods E R. Digital Image Processing. NY: Pearson; 2018.

[7] Pitie F, Kokaram A. The linear Monge-Kantorovitch linear colour mapping for example-based colour transfer. IET 4th European Conference on Visual Media Production (CVMP 2007). London, UK: IEE; 2007. pp. 23–23. doi: 10.1049/cp:20070055

[8] Hahne C, Aggoun A. PlenoptiCam v1.0: A Light-Field Imaging Framework. IEEE Transactions on Image Processing. 2021;30: 6757–6771. doi: 10.1109/TIP.2021.3095671 34280098

[9] Shamey R, Brill MH. McCamy Calvin S. 1924–2017. Pioneers of Color Science. Cham: Springer International Publishing; 2020. pp. 405–407. doi: 10.1007/978-3-319-30811-1_92

[10] McCamy CS, Marcus H, Davidson JG. A color-rendition chart. J App Photog Eng. 1976;2: 95–99.

[11] Sharma G, Wu W, Dalal EN. The CIEDE2000 color-difference formula: Implementation notes, supplementary test data, and mathematical observations. Color Res Appl. 2005;30: 21–30. doi: 10.1002/col.20070

[12] E12 Committee. Practice for Specifying and Verifying the Performance of Color-Measuring Instruments. ASTM International; doi: 10.1520/E2214-20

[13] Tan M, Le QV. EfficientNetV2: Smaller Models and Faster Training. 2021 [cited 22 May 2024]. doi: 10.48550/ARXIV.2104.00298

[14] Abadi M, Agarwal A, Barham P, Brevdo E, Chen Z, Citro C, et al. TensorFlow: Large-Scale Machine Learning on Heterogeneous Distributed Systems. 2016.

[15] Evaluation of Precision Performance of Quantitative Measurement Methods (EP05-A3). Clinical and Laboratory Standards Institute. CLSI; 2014.

[16] Evaluation of Detection Capability for Clinical Laboratory Measurement Procedures (EP17-A2). Clinical and Laboratory Standards Institute. CLSI; 2012.

[17] Evaluation of the Linearity of Quantitative Measurement Procedures (EP6-A). Clinical and Laboratory Standards Institute. CLSI;

[18] Vashist SK, Van Oordt T, Schneider EM, Zengerle R, Von Stetten F, Luong JHT. A smartphone-based colorimetric reader for bioanalytical applications using the screen-based bottom illumination provided by gadgets. Biosensors and Bioelectronics. 2015;67: 248–255. doi: 10.1016/j.bios.2014.08.027 25168283

[19] Liu J, Geng Z, Fan Z, Liu J, Chen H. Point-of-care testing based on smartphone: The current state-of-the-art (2017–2018). Biosensors and Bioelectronics. 2019;132: 17–37. doi: 10.1016/j.bios.2019.01.068 30851493

[20] Hunt B, Ruiz AJ, Pogue BW. Smartphone-based imaging systems for medical applications: a critical review. J Biomed Opt. 2021;26. doi: 10.1117/1.JBO.26.4.040902 33860648 PMC8047775

[21] Wang S, Wang H, Ding Y, Li W, Gao H, Ding Z, et al. Filter paper- and smartphone-based point-of-care tests for rapid and reliable detection of artificial food colorants. Microchemical Journal. 2022;183: 108088. doi: 10.1016/j.microc.2022.108088

[22] Coleman B, Coarsey C, Kabir MA, Asghar W. Point-of-care colorimetric analysis through smartphone video. Sensors and Actuators B: Chemical. 2019;282: 225–231. doi: 10.1016/j.snb.2018.11.036 30828133 PMC6391882

