# Supplementary material for: Development of a smartphone enabled, paper-based quantitative diagnostic assay using the HueDx color correction system
Source: PLoS One. 2024 Oct 4;19(10):e0311343. doi: 10.1371/journal.pone.0311343 (PMC11451979; doi:10.1371/journal.pone.0311343)
Supplement: S1 File — (PDF) [file pone.0311343.s001.pdf]

Development of a smartphone enabled, paper-based quantitative diagnostic assay using the HueDx color correction system

Nidhi Menon[1]*, David Beery[1], Prava Sharma[1], Adrian Crutchfield[1], Leah Kim[1], Aaron Lauer[1], Ayesha Azimuddin[1], Brianna Wronko-Stevens[1]

*nidhi.menon@huedx.com

1. HueDx,Inc., Philadelphia, Pennsylvania, United States of America

Supporting Information.

Table 1. $\Delta E00$ Mean at a color patch level of the sticker

Color Patch Number	$\Delta E00$ Mean	$\Delta E00$ Max	Standard Deviation
1	0.70	1.66	0.38
2	0.99	2.38	0.55
3	1.91	4.35	0.85
4	1.84	4.59	0.91
5	2.14	4.57	1.02
6	1.75	4.17	0.89
7	1.46	4.12	0.93
8	1.64	3.48	0.79
9	1.88	5.08	1.08
10	2.08	5.07	1.21
11	2.68	5.28	1.42
12	1.75	3.95	1.05
13	1.86	3.98	1.10
14	1.33	2.99	0.65
15	1.67	4.22	0.82
16	2.27	5.39	1.19
17	1.81	4.50	0.91

18	1.75	4.42	1.03
19	1.70	3.88	1.01
20	2.46	5.64	1.41
21	1.75	4.07	0.85
22	2.31	5.38	1.42
23	2.57	5.61	1.57
24	2.59	5.10	1.51
25	1.69	3.35	0.73
26	1.29	246	0.60
**MEAN:**	**1.84**	**4.22**	**1.00**

Table 2. The average, max $\Delta E00$ and standard deviation score for each sticker patch across all the illumination conditions.

Illumination Temperature	Intra-Site $\Delta E00$Mean	$\Delta E00$ Mean from Ground Truth	$\Delta E00$ Max from Ground Truth	Standard Deviation
~5800K	2.09	4.66	7.80	1.53
5000K	0.81	14.96	21.09	4.40
4000K	1.05	16.25	23.93	4.55
3000K	1.19	9.42	17.33	3.03
2700K	0.81	18.30	26.65	5.29
**MEAN:**	**1.19**	**12.72**	**19.36**	**6.39**

Table 3. The inter-site average, max $\Delta E00$ and standard deviation score for each sticker patch pre-correction

Color Patch Number	$\Delta E00$ Mean	$\Delta E00$ Max	Standard Deviation

1	1.73	3.42	0.97
2	2.61	5.09	1.40
3	6.67	14.95	4.27
4	8.79	20.39	5.71
5	10.52	19.58	5.91
6	8.29	13.24	4.10
7	8.03	18.44	5.63
8	6.24	13.64	4.35
9	9.75	18.54	5.54
10	10.24	20.87	6.89
11	10.88	20.51	6.85
12	7.56	14.19	4.48
13	7.27	15.71	4.23
14	7.11	16.80	4.62
15	6.54	14.92	4.89
16	9.30	18.98	6.46
17	7.33	18.24	5.34
18	9.72	24.33	6.94
19	7.34	18.04	5.14
20	9.86	17.33	6.07
21	7.79	17.64	4.79
22	10.76	22.41	6.35
23	10.28	20.25	6.80
24	11.20	23.02	6.95
25	7.72	14.53	3.82
26	1.78	3.33	0.91
**MEAN:**	**7.90**	**16.48**	**4.98**

Table 4. The inter-site average, max $\Delta E00$ Mean and standard deviation score for each sticker patch post-correction

Color Patch Number	$\Delta E00$ Mean	$\Delta E00$ Max	Standard Deviation
1	2.25	4.83	1.09
2	2.53	5.22	1.20
3	2.11	4.05	0.97
4	1.93	3.67	0.88
5	1.68	3.81	0.70
6	1.99	3.81	0.99
7	1.83	4.22	1.34
8	1.76	3.43	0.74
9	1.36	2.82	0.63
10	2.19	3.90	1.30
11	2.74	7.40	1.76
12	1.83	4.35	1.18
13	1.82	3.61	0.90
14	2.20	5.17	1.64
15	1.74	3.55	0.85
16	2.14	4.70	1.53
17	1.79	3.63	0.85
18	2.52	5.97	1.54
19	2.33	5.59	1.44
20	1.57	3.76	0.96
21	1.96	4.18	1.30
22	1.32	2.48	0.63

23	1.53	3.66	0.95
24	2.57	5.63	1.56
25	4.21	9.50	2.55
26	1.98	4.76	1.08
**MEAN:**	**2.07** (↓74%)	**4.53** (↓73%)	**1.18** (↓55%)

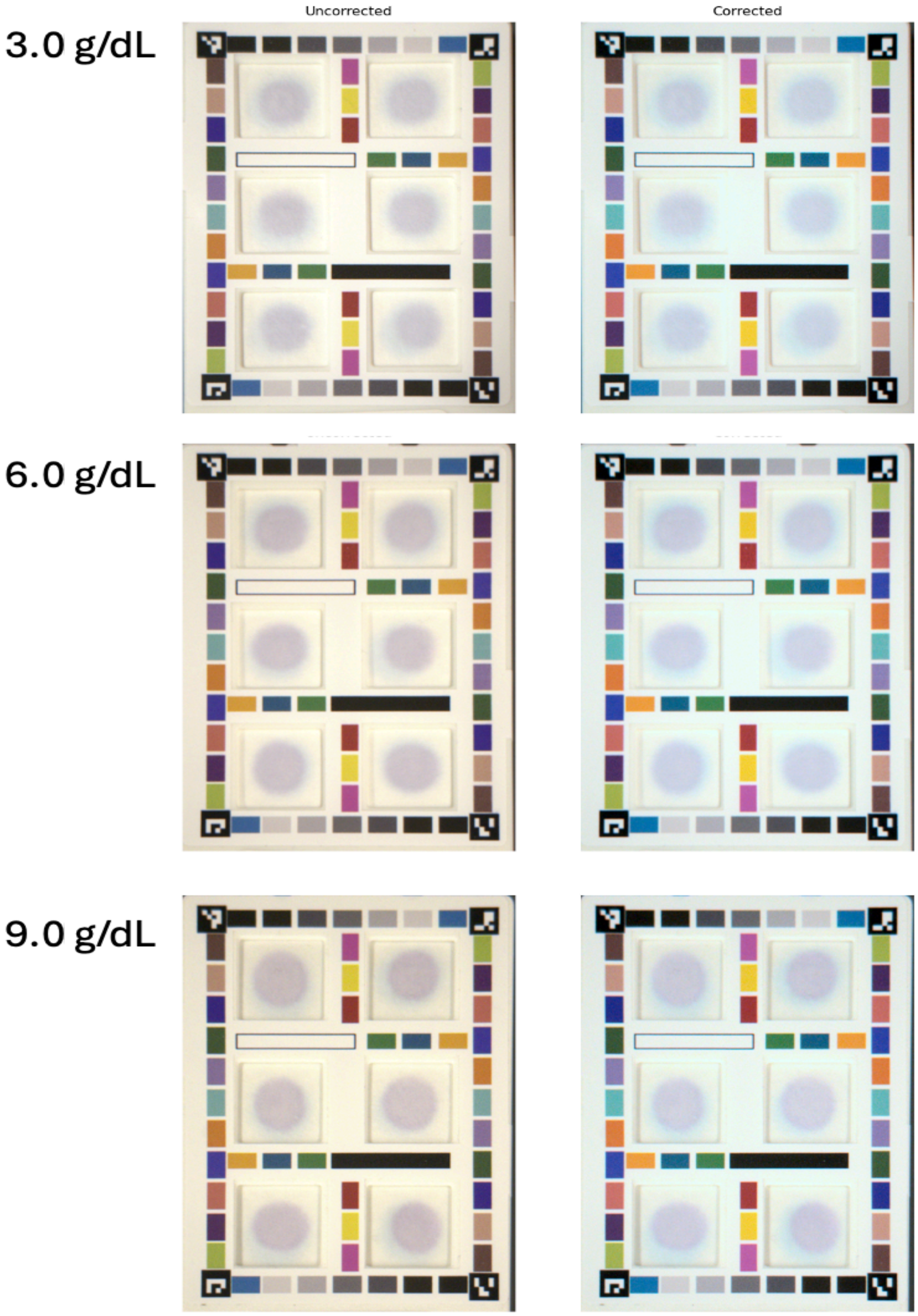

Fig 1. Uncorrected and corrected images of end-point total protein concentrations showing the variation in color under ambient lighting when uncorrected and the color-correction carried out to standardize it.

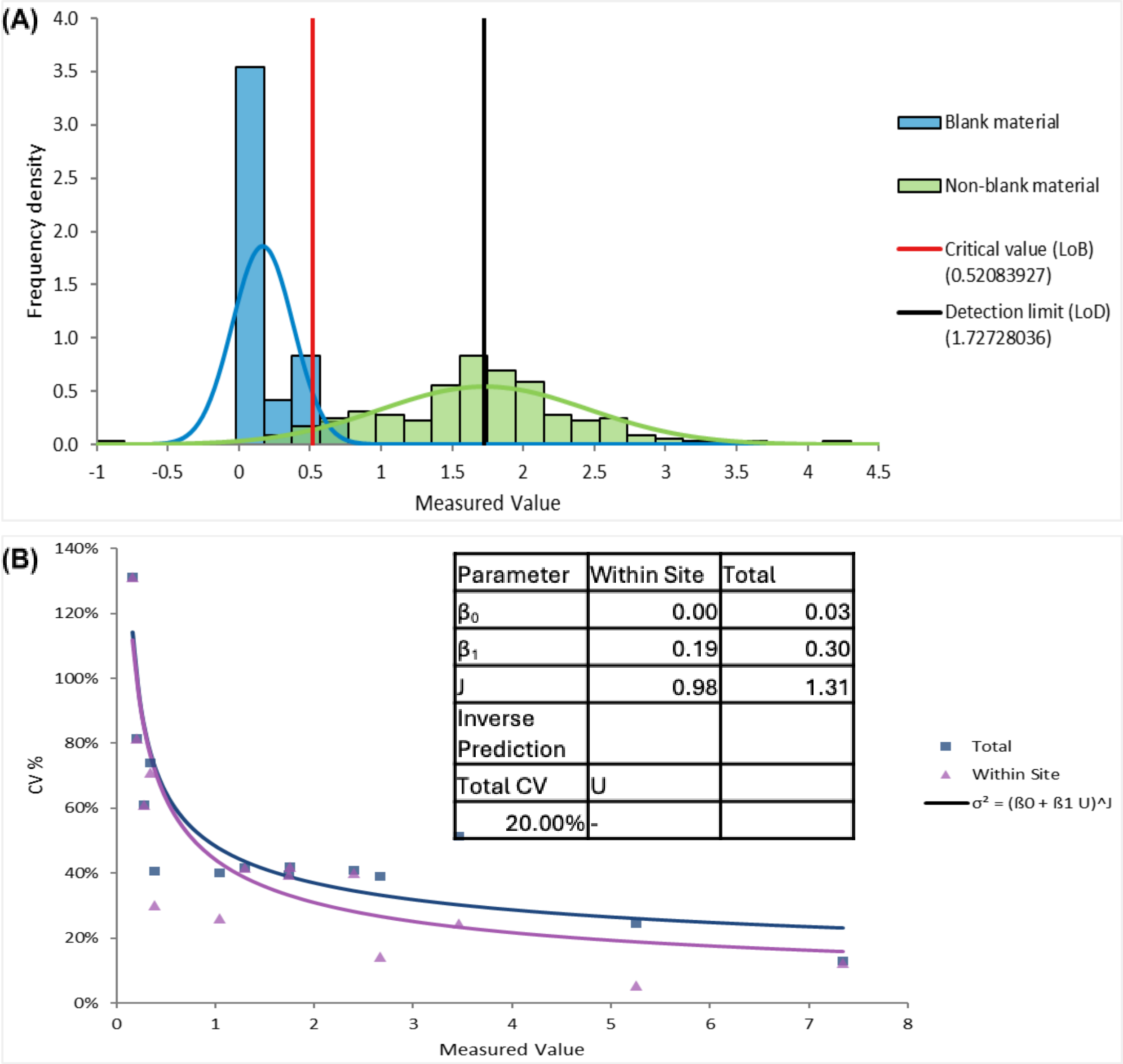

Fig 2. Limits of detection for uncorrected images. (A) The limits of blank and detection calculated using the normal quantile distribution of the predictions with known values of samples. **(B)** The %CV precision profile and the variance function describing the profile. The limit of quantitation was unable to be determined due to the poor performance of the variance function.

Table 5 (A). Model Fit Parameters for linear model, 2nd and 3rd order polynomial for the predictions from the color corrected model.

Linear fit					
RMSE	0.619976735				
Parameter	Estimate	SE	t	DF	p-value
Constant	-0.1377	0.064335	-2.14	202	0.0335
X	1.061	0.017435	3.50	202	0.0006
Source	SS	DF	MS	F	p-value
Lack of fit	40.721315445	12	3.393442954	17.46	<0.0001
Pure error	36.921657289	190	0.194324512		
Model error	77.642972735	202	0.384371152		
2nd order polynomial fit					
RMSE	0.620676831				
Parameter	Estimate	SE	t	DF	p-value
Constant	-0.1959	0.10186	-1.92	201	0.0558
X	1.110	0.069114	1.60	201	0.1121
X^2	-0.005594	7.5811E-03	-0.74	201	0.4614
Source	SS	DF	MS	F	p-value
Lack of fit	40.511528072	11	3.682866188	18.95	<0.0001
Pure error	36.921657289	190	0.194324512		
Model error	77.433185361	201	0.385239728		
3rd order polynomial fit					
RMSE	0.616953450				
Parameter	Estimate	SE	t	DF	p-value
Constant	-0.08688	0.11711	-0.74	200	0.4590
X	0.9324	0.11804	-0.57	200	0.5677
X^2	0.05518	0.033653	1.64	200	0.1026
X^3	-0.004927	2.6591E-03	-1.85	200	0.0654
Source	SS	DF	MS	F	p-value
Lack of fit	39.204654717	10	3.920465472	20.17	<0.0001
Pure error	36.921657289	190	0.194324512		
Model error	76.126312006	200	0.380631560		

Table 5 (B). Model Fit Parameters for linear model, 2nd and 3rd order polynomial for the predictions from the non color-corrected model.

Linear fit					
RMSE	0.856541697				
Parameter	Estimate	SE	t	DF	p-value
Constant	-0.1132	0.088883	-1.27	202	0.2043
X	0.7351	0.024088	-11.00	202	<0.0001
Source	SS	DF	MS	F	p-value
Lack of fit	57.574858930	12	4.797904911	10.06	<0.0001
Pure error	90.625204272	190	0.476974759		
Model error	1.482001E+02	202	0.733663679		
2nd order polynomial fit					
RMSE	0.848179762				
Parameter	Estimate	SE	t	DF	p-value
Constant	0.1280	0.13919	0.92	201	0.3589
X	0.5307	0.094447	-4.97	201	<0.0001
X^2	0.02317	0.010360	2.24	201	0.0264 [1]

[1] Nonlinear parameter is different from 0 at the 5% significance level.

Source	SS	DF	MS	F	p-value
Lack of fit	53.975986437	11	4.906907858	10.29	<0.0001
Pure error	90.625204272	190	0.476974759		
Model error	1.446012E+02	201	0.719408909		
3rd order polynomial fit					
RMSE	0.816131311				
Parameter	Estimate	SE	t	DF	p-value
Constant	-0.1939	0.15491	-1.25	200	0.2122
X	1.056	0.15615	0.36	200	0.7215
X^2	-0.1562	0.044518	-3.51	200	0.0006 [1]
X^3	0.01454	3.5176E-03	4.13	200	<0.0001 [1]

[1] Nonlinear parameter is different from 0 at the 5% significance level.

Table 6. Lux measurements taken at each site and their corresponding color temperatures.

Color Temperature	Illuminance (lux)	ΔE00 Mean Pre Correction	ΔE00 Mean Post Correction
~5800k	8400	4.66	3.07
5000k	378	14.96	3.82
4000k	140	16.25	3.51
3000k	85	9.42	3.89
2700k	26	18.30	4.79

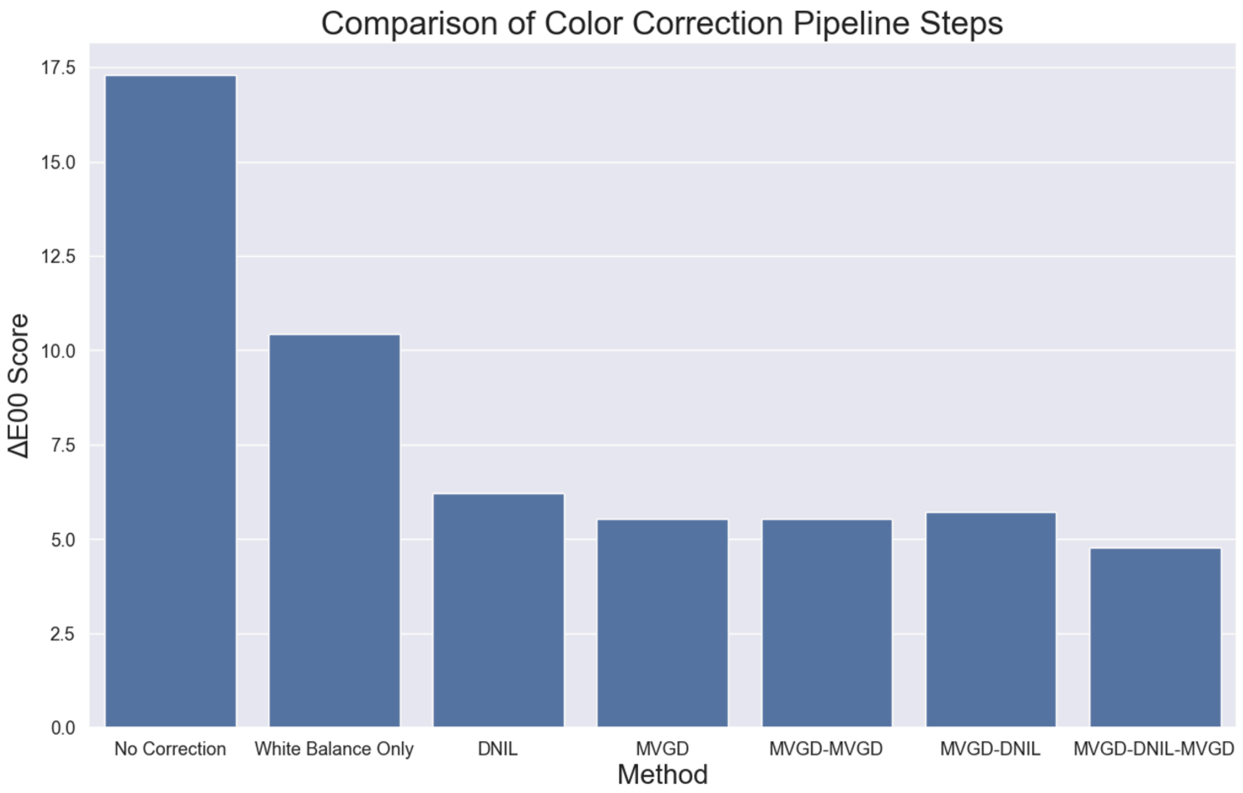

Fig 3. The ablation experiment shows the need for the color correction steps and order of the steps selected in the study.
