# Supplementary figures and images for: Development of a smartphone enabled, paper-based quantitative diagnostic assay using the HueDx color correction system
Source: PLoS One. 2024 Oct 4;19(10):e0311343. doi: 10.1371/journal.pone.0311343 (PMC11451979; doi:10.1371/journal.pone.0311343)

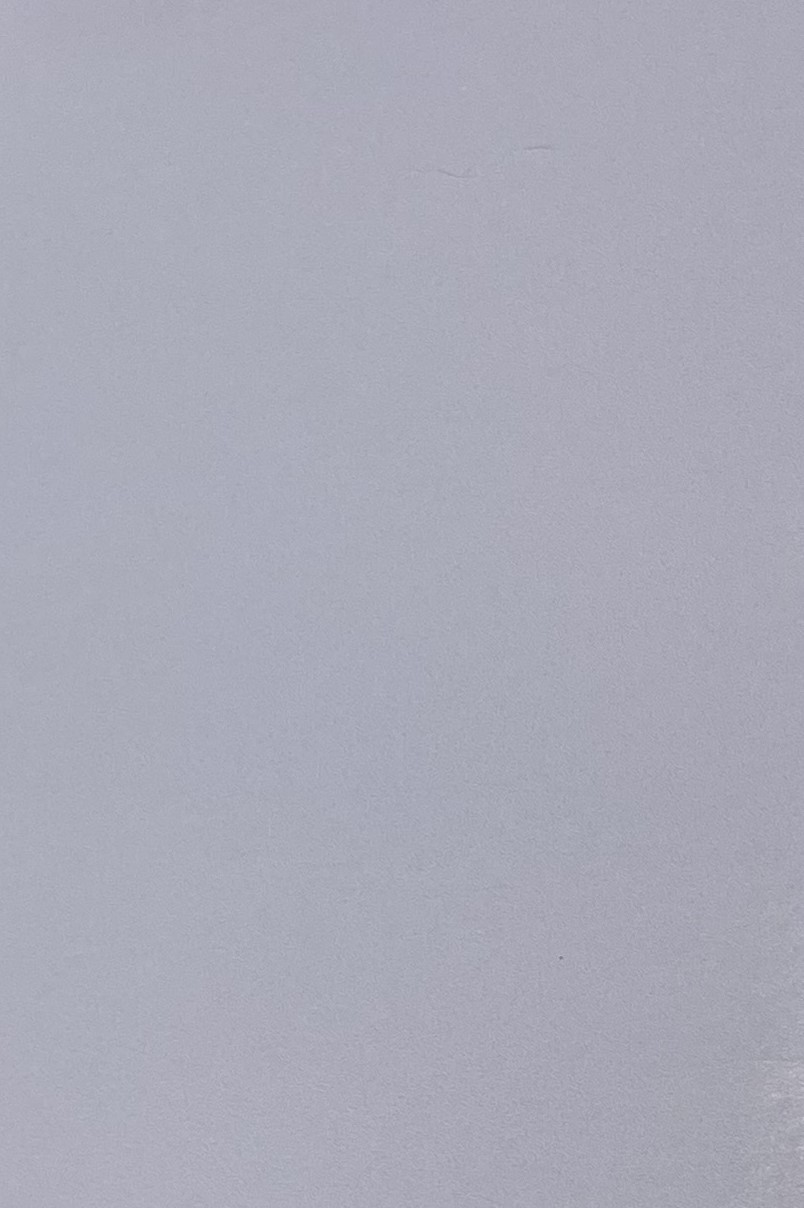

Supplement: S2 File — (ZIP) [file pone.0311343.s002.zip › S2 File/White (4).JPG]

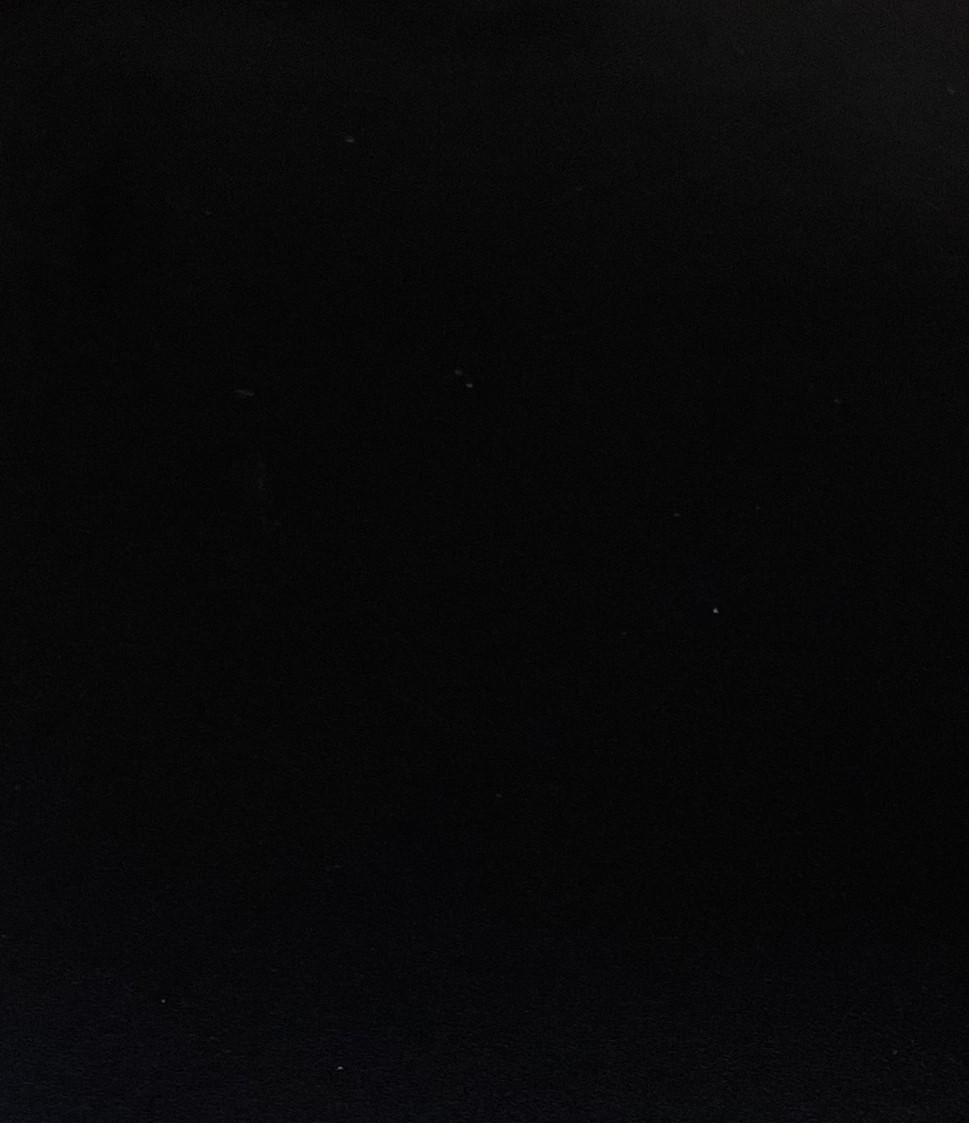

Supplement: S2 File — (ZIP) [file pone.0311343.s002.zip › S2 File/Black (8).JPG]

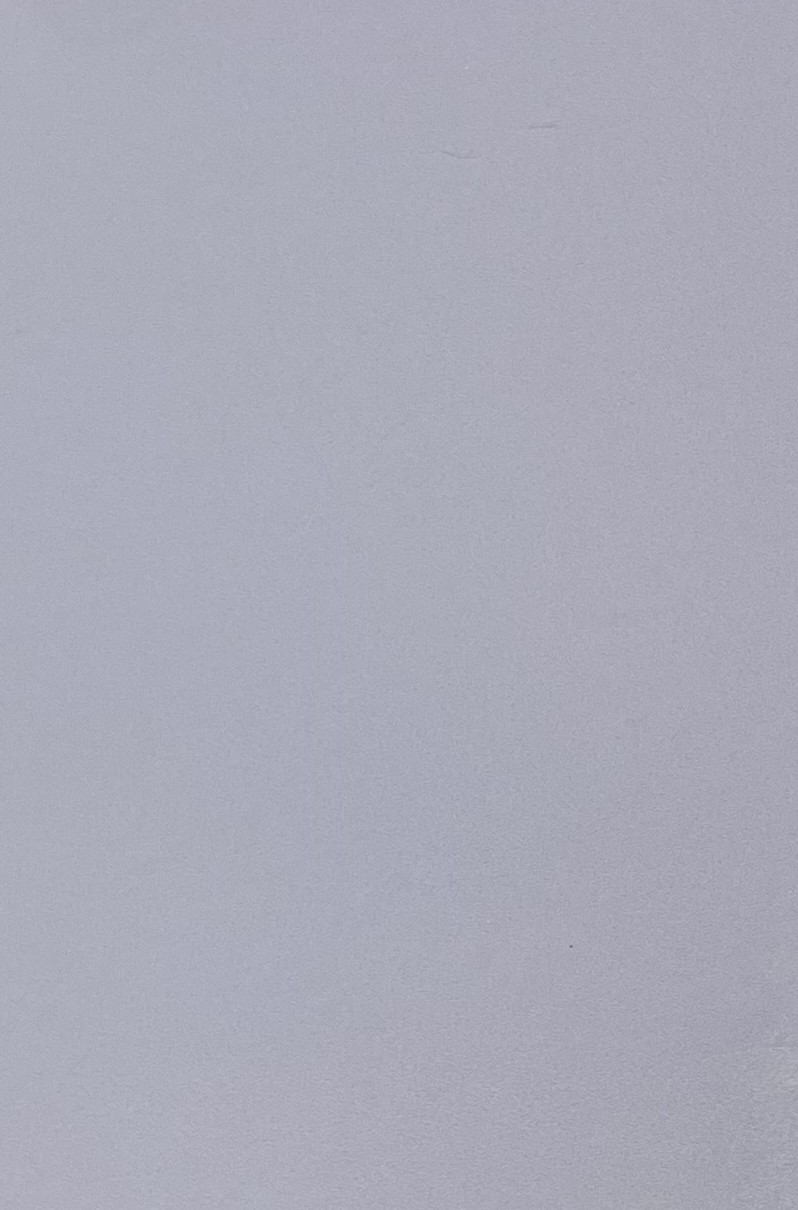

Supplement: S2 File — (ZIP) [file pone.0311343.s002.zip › S2 File/White (8).JPG]

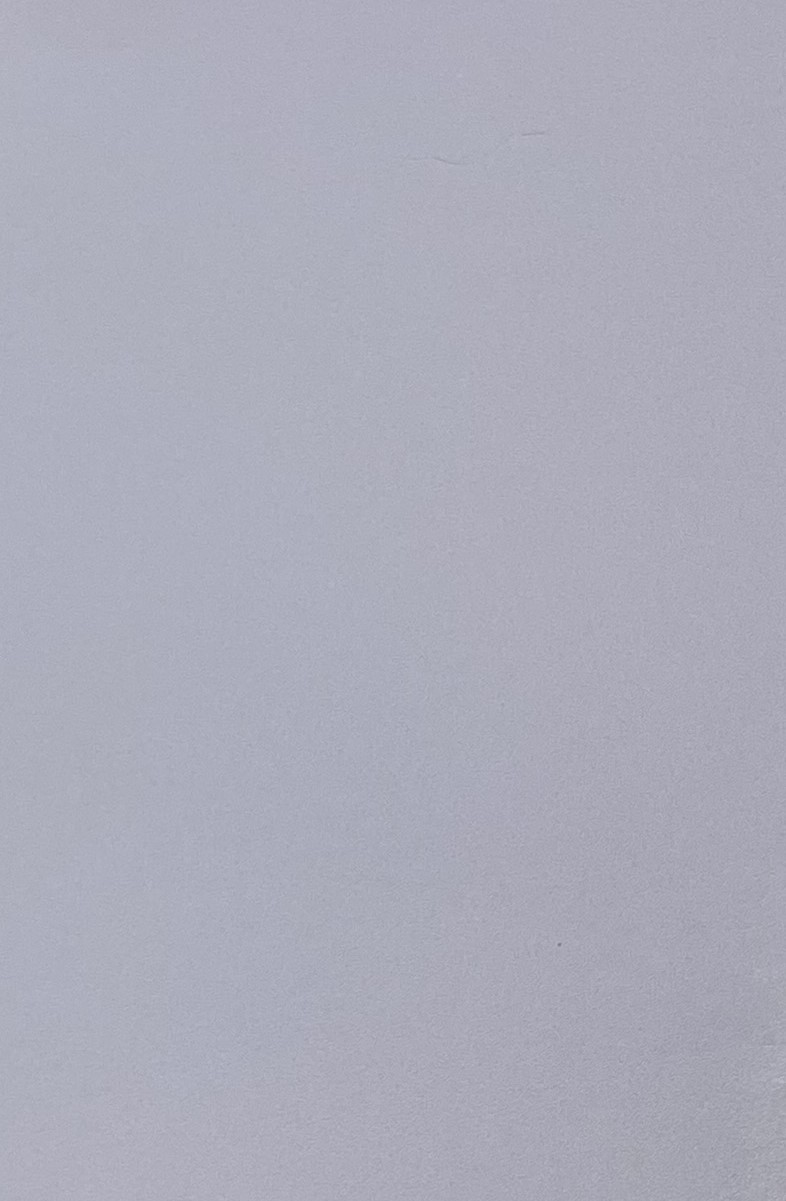

Supplement: S2 File — (ZIP) [file pone.0311343.s002.zip › S2 File/White (10).JPG]

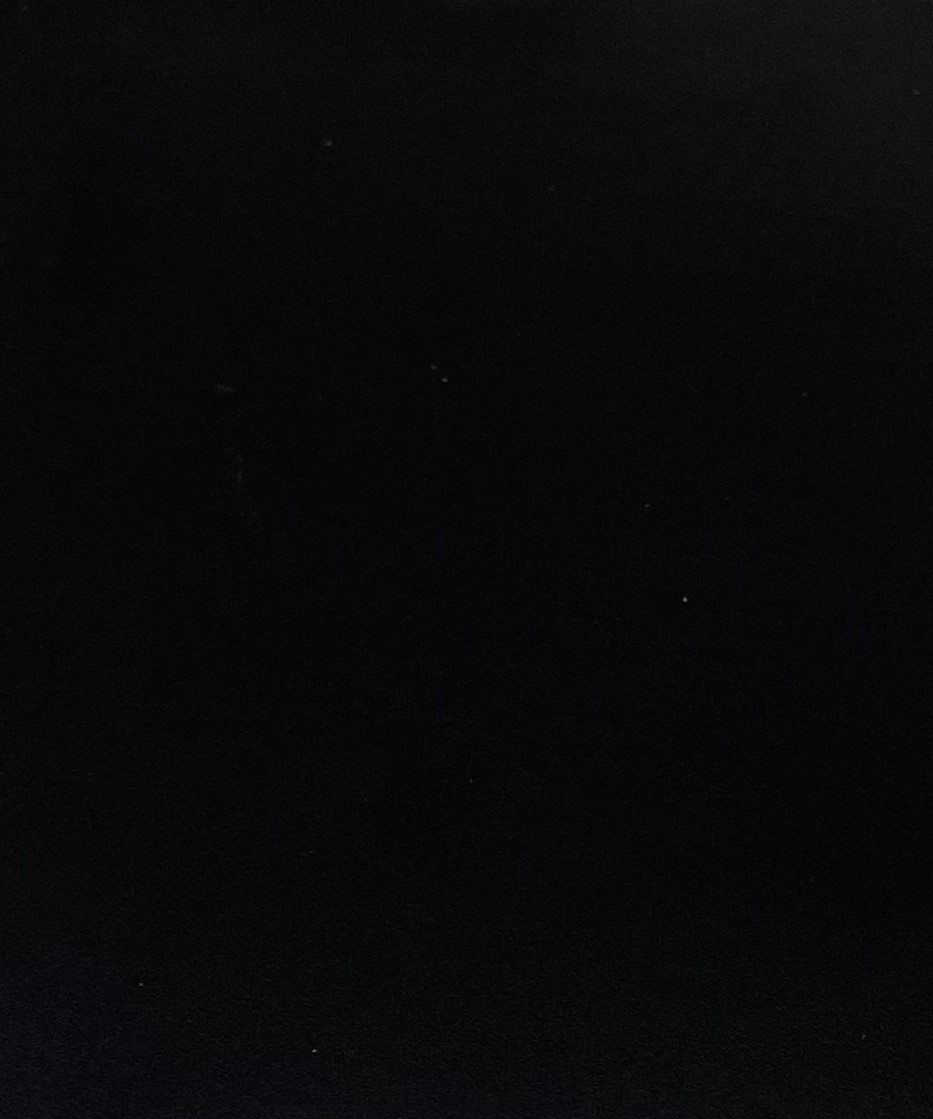

Supplement: S2 File — (ZIP) [file pone.0311343.s002.zip › S2 File/Black (4).JPG]

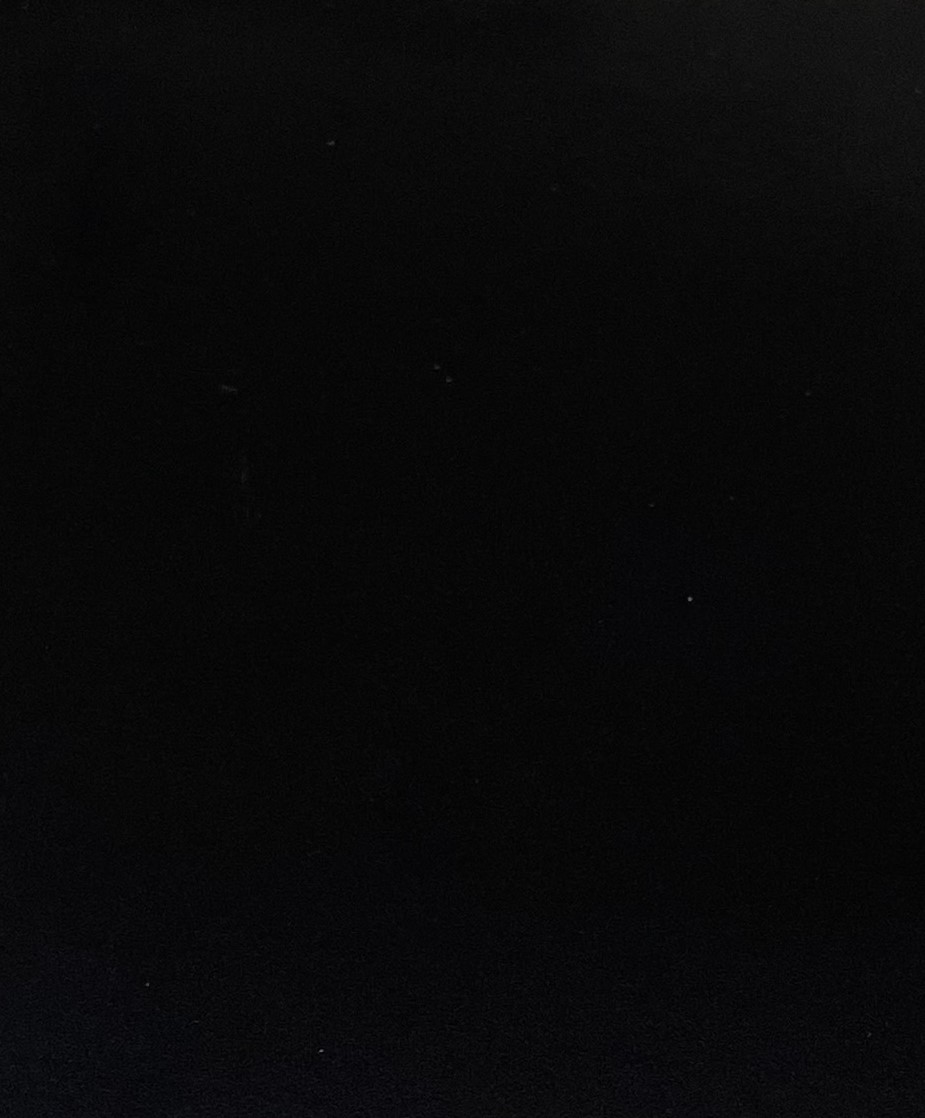

Supplement: S2 File — (ZIP) [file pone.0311343.s002.zip › S2 File/Black (5).JPG]

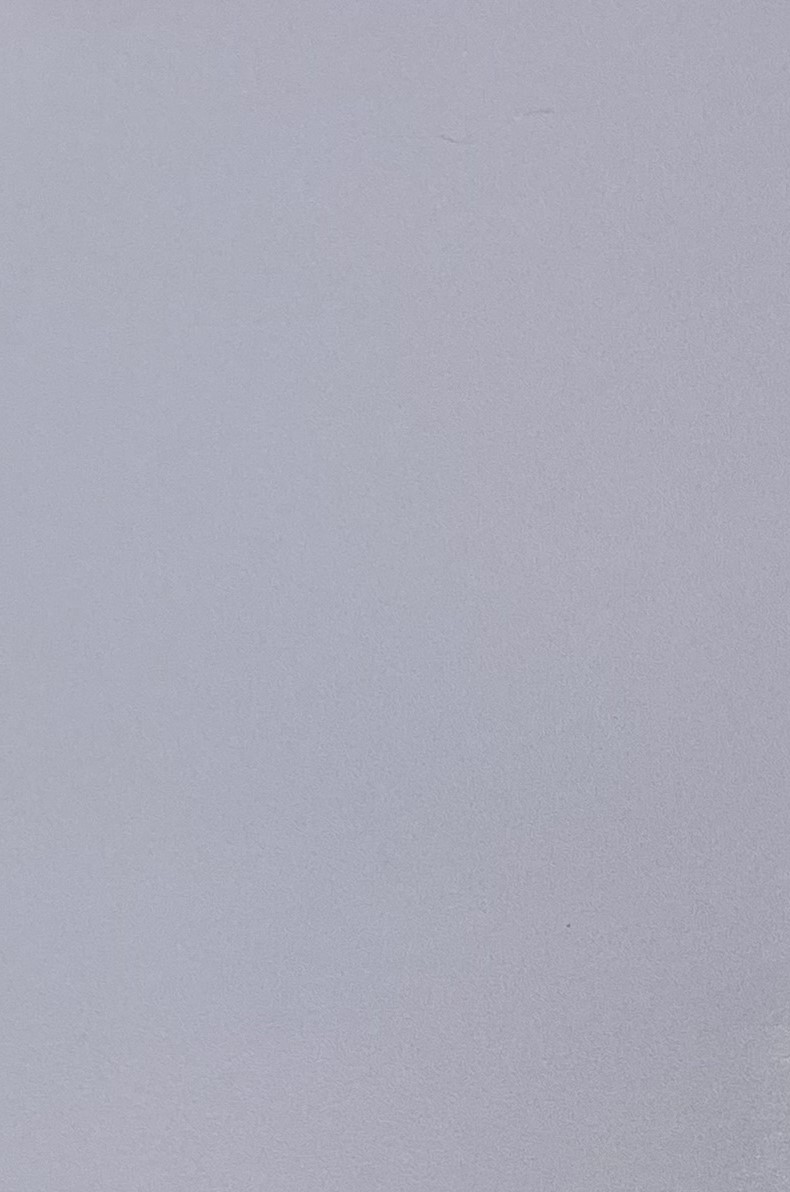

Supplement: S2 File — (ZIP) [file pone.0311343.s002.zip › S2 File/White (9).JPG]

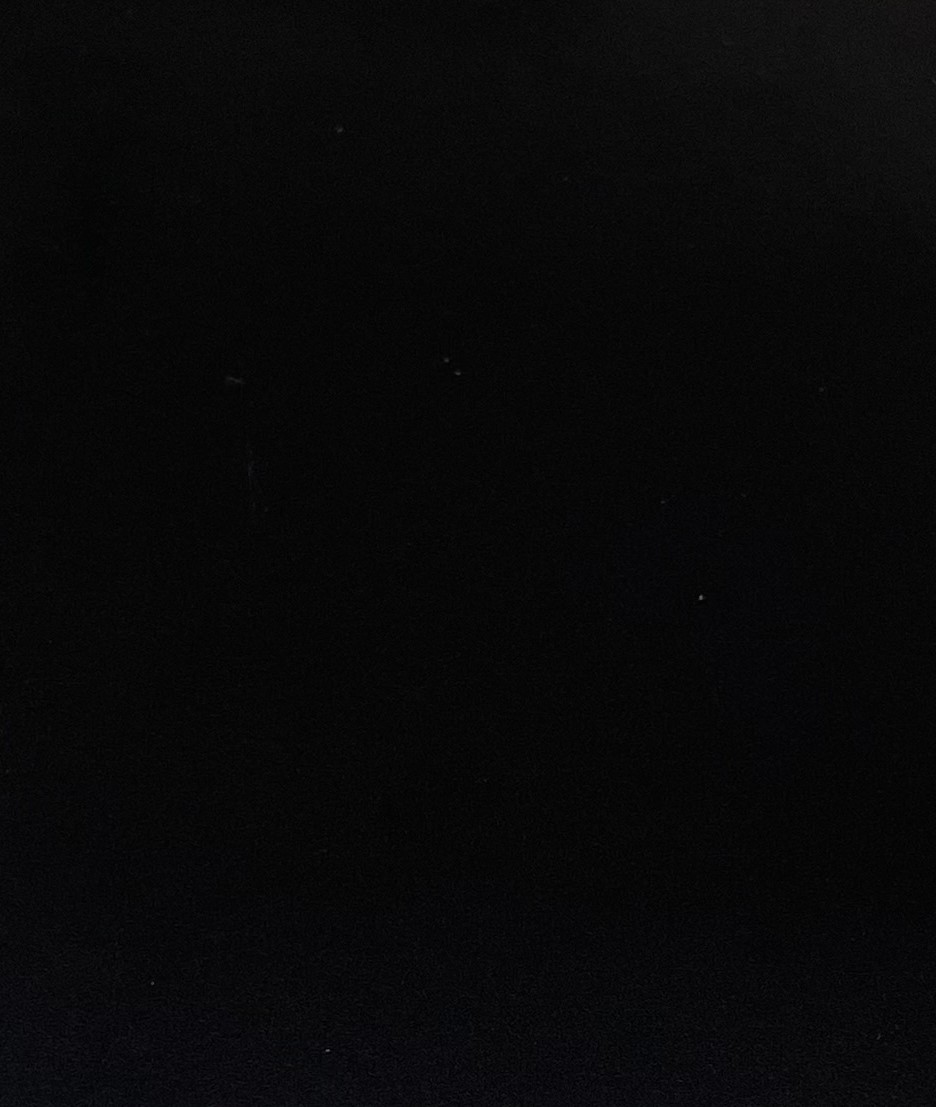

Supplement: S2 File — (ZIP) [file pone.0311343.s002.zip › S2 File/Black (9).JPG]

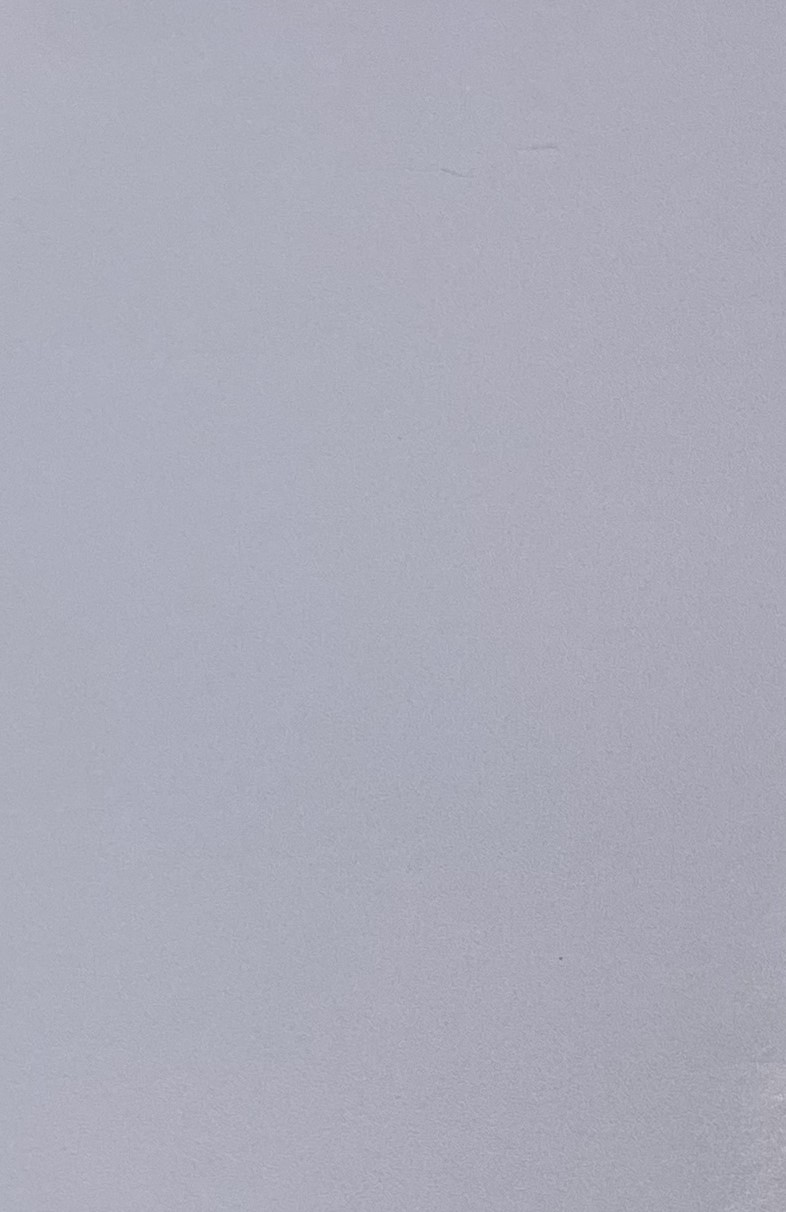

Supplement: S2 File — (ZIP) [file pone.0311343.s002.zip › S2 File/White (5).JPG]

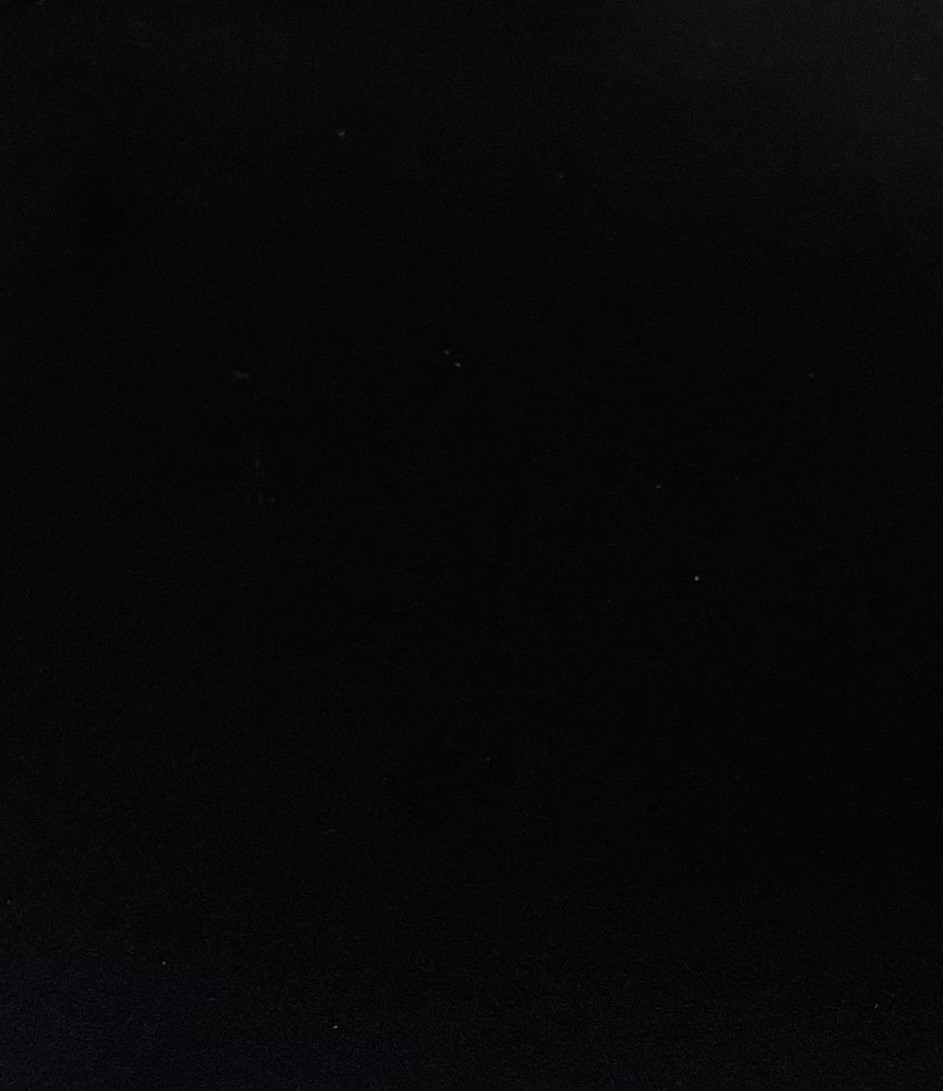

Supplement: S2 File — (ZIP) [file pone.0311343.s002.zip › S2 File/Black (2).JPG]

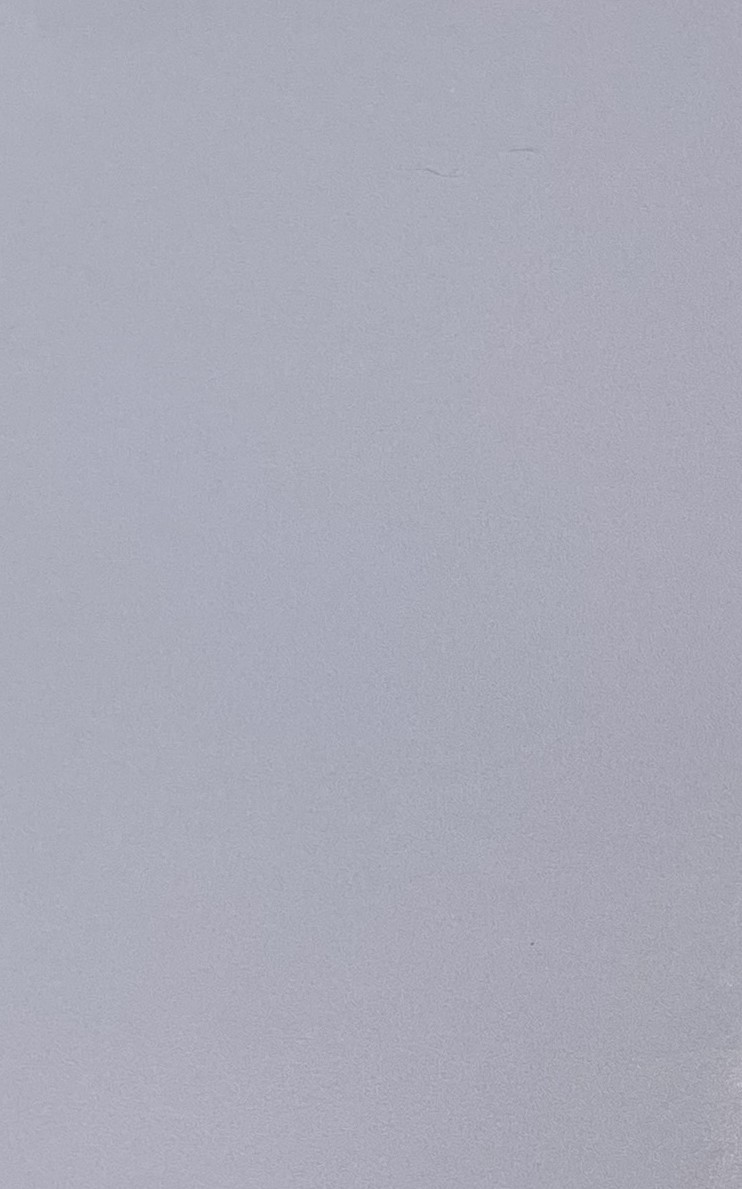

Supplement: S2 File — (ZIP) [file pone.0311343.s002.zip › S2 File/White (2).JPG]

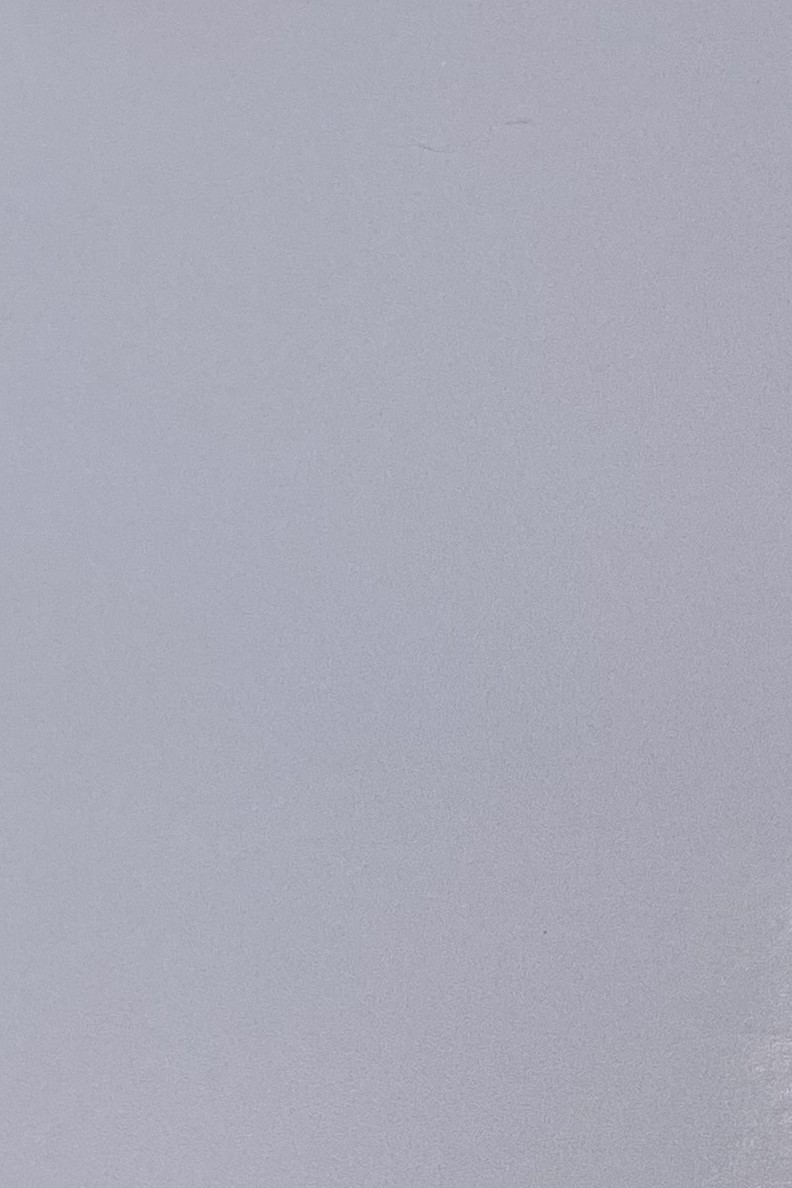

Supplement: S2 File — (ZIP) [file pone.0311343.s002.zip › S2 File/White (3).JPG]

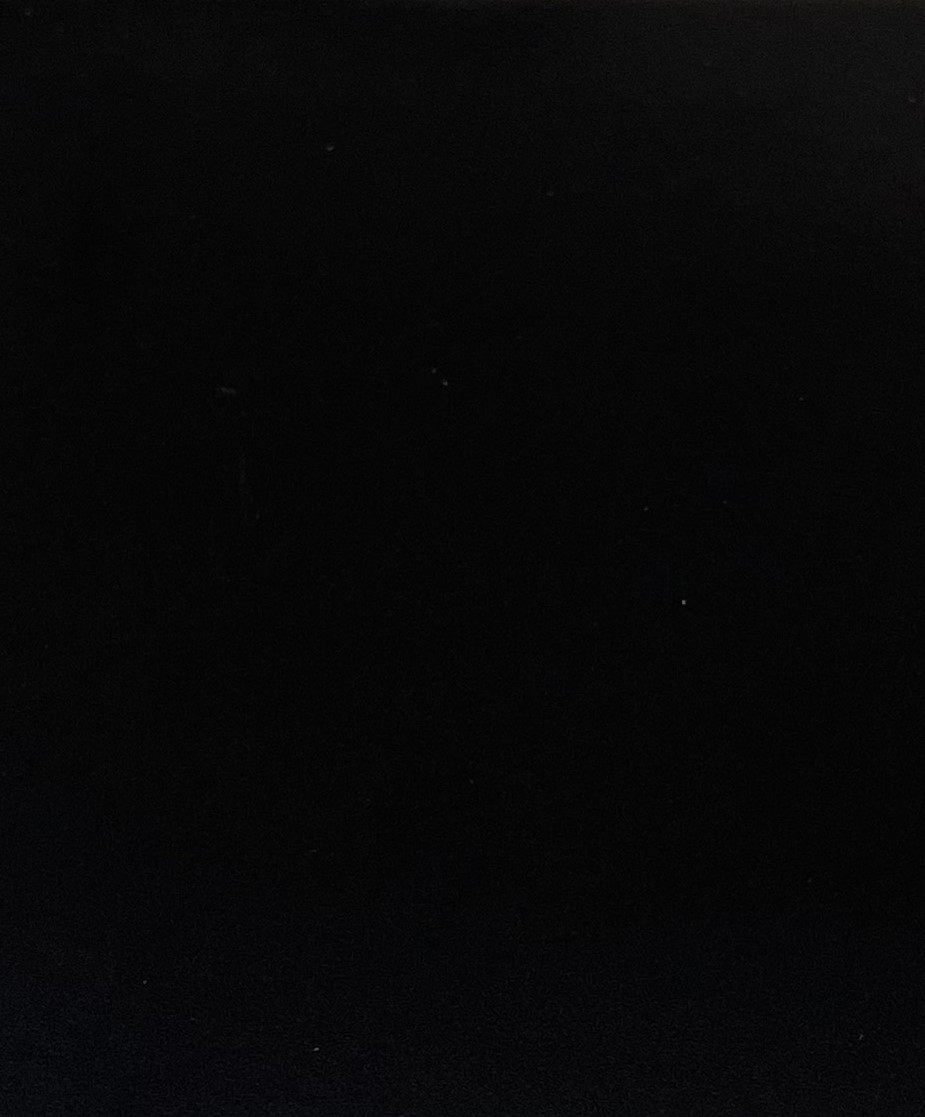

Supplement: S2 File — (ZIP) [file pone.0311343.s002.zip › S2 File/Black (3).JPG]

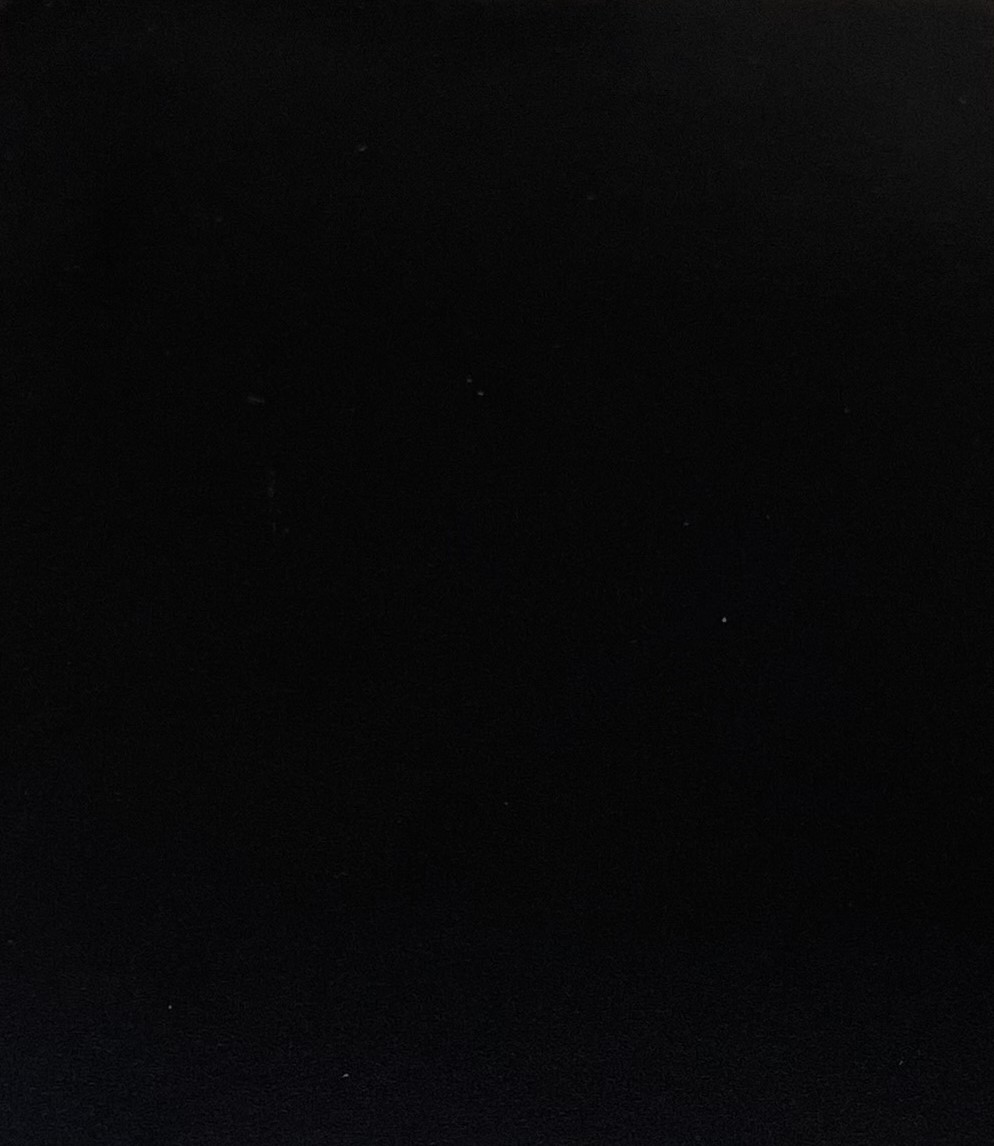

Supplement: S2 File — (ZIP) [file pone.0311343.s002.zip › S2 File/Black (10).JPG]

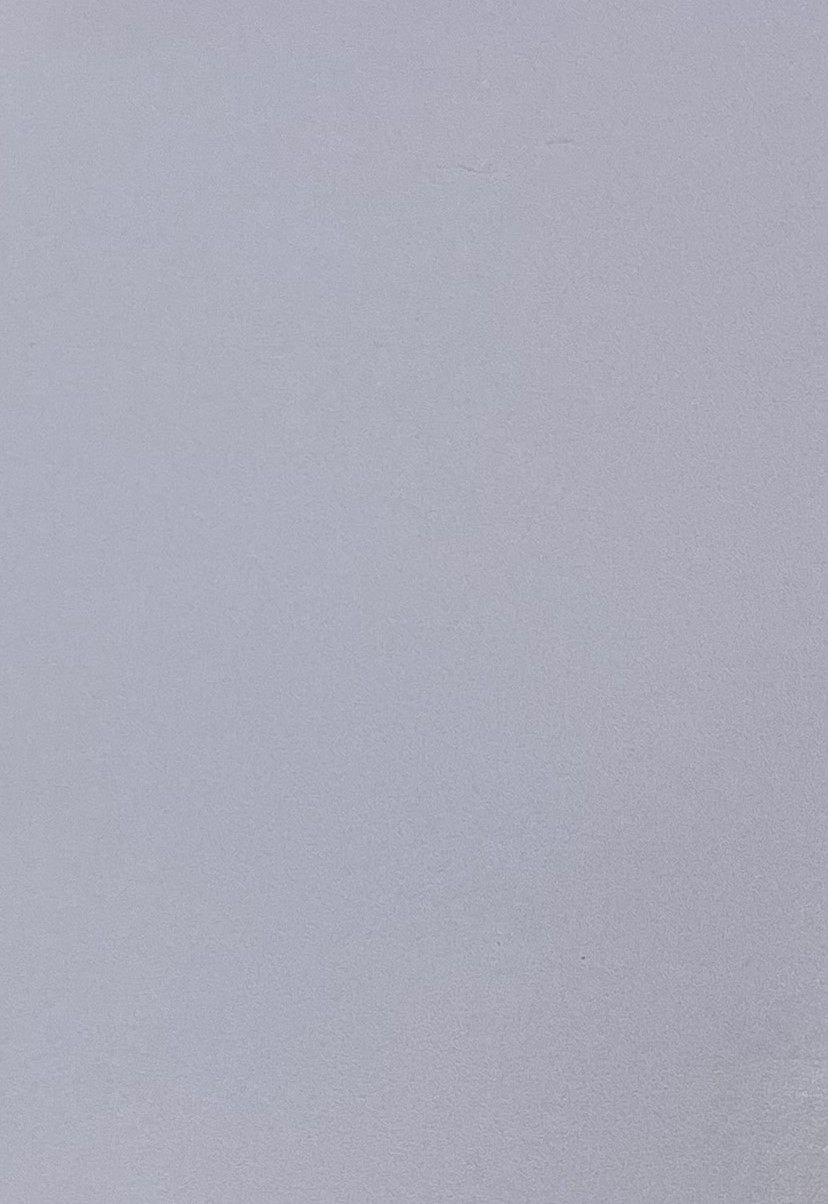

Supplement: S2 File — (ZIP) [file pone.0311343.s002.zip › S2 File/White (1).JPG]

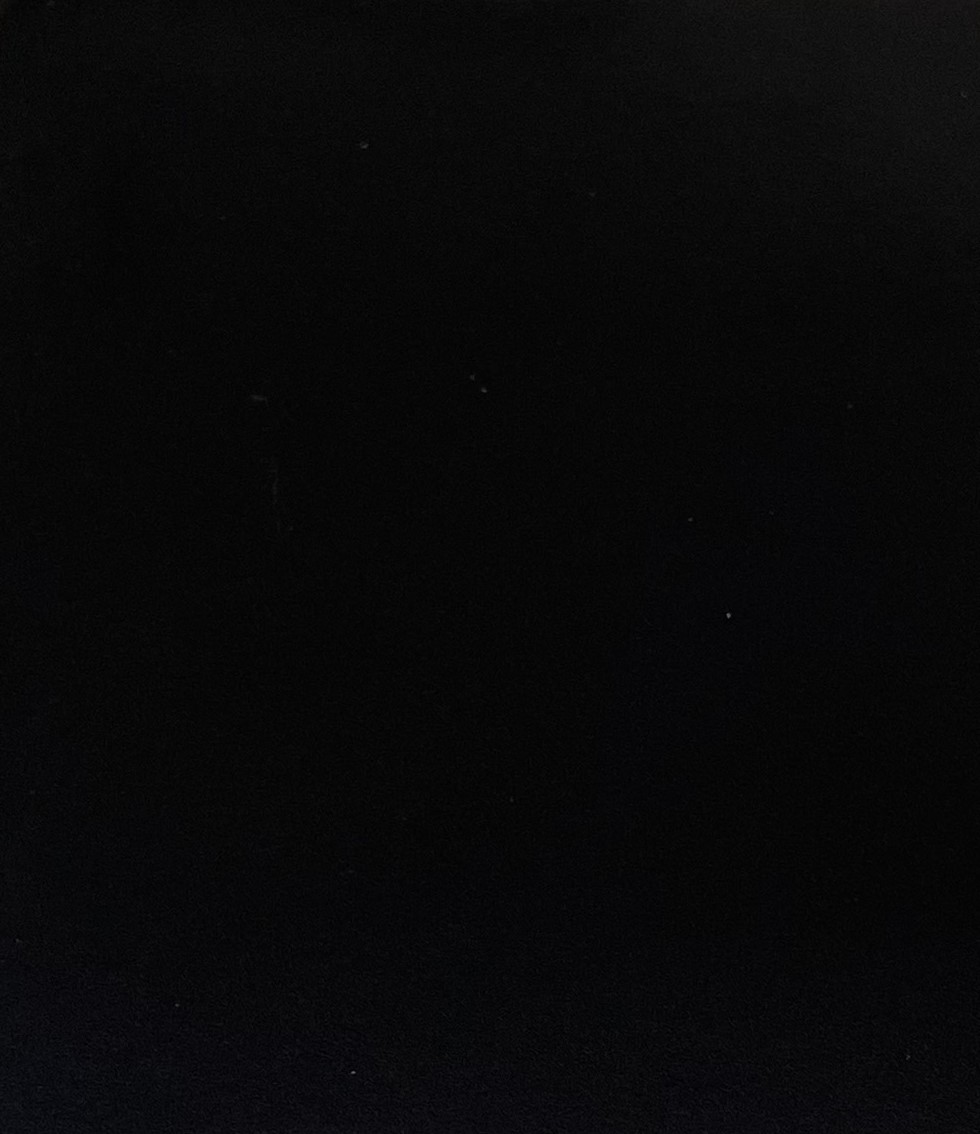

Supplement: S2 File — (ZIP) [file pone.0311343.s002.zip › S2 File/Black (1).JPG]

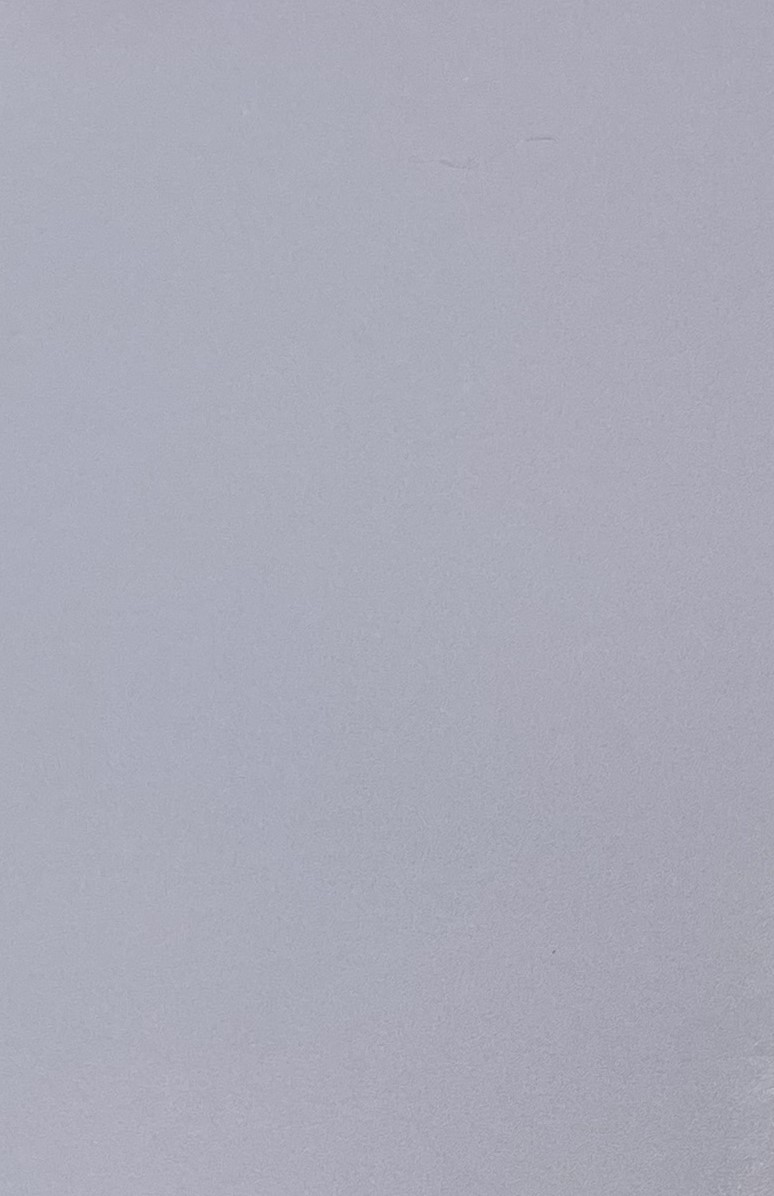

Supplement: S2 File — (ZIP) [file pone.0311343.s002.zip › S2 File/White (6).JPG]

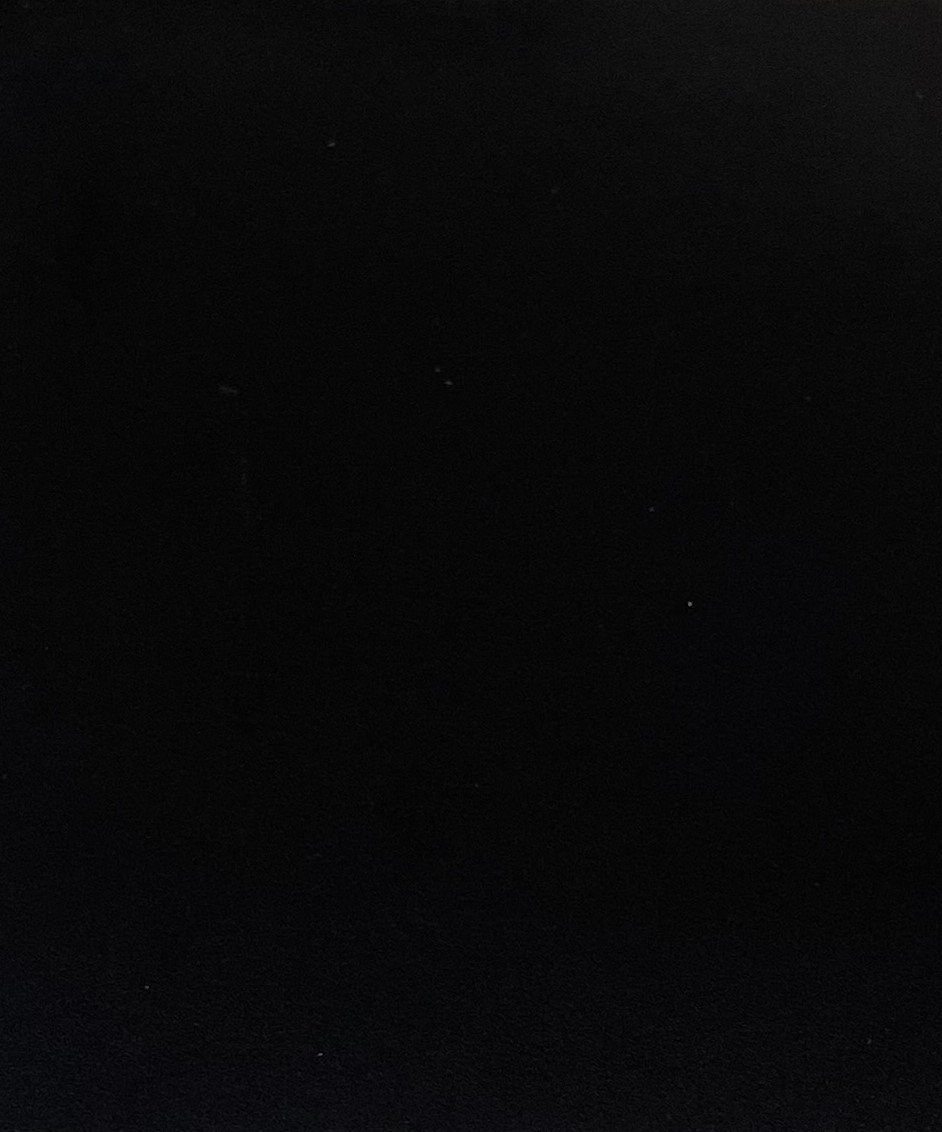

Supplement: S2 File — (ZIP) [file pone.0311343.s002.zip › S2 File/Black (6).JPG]

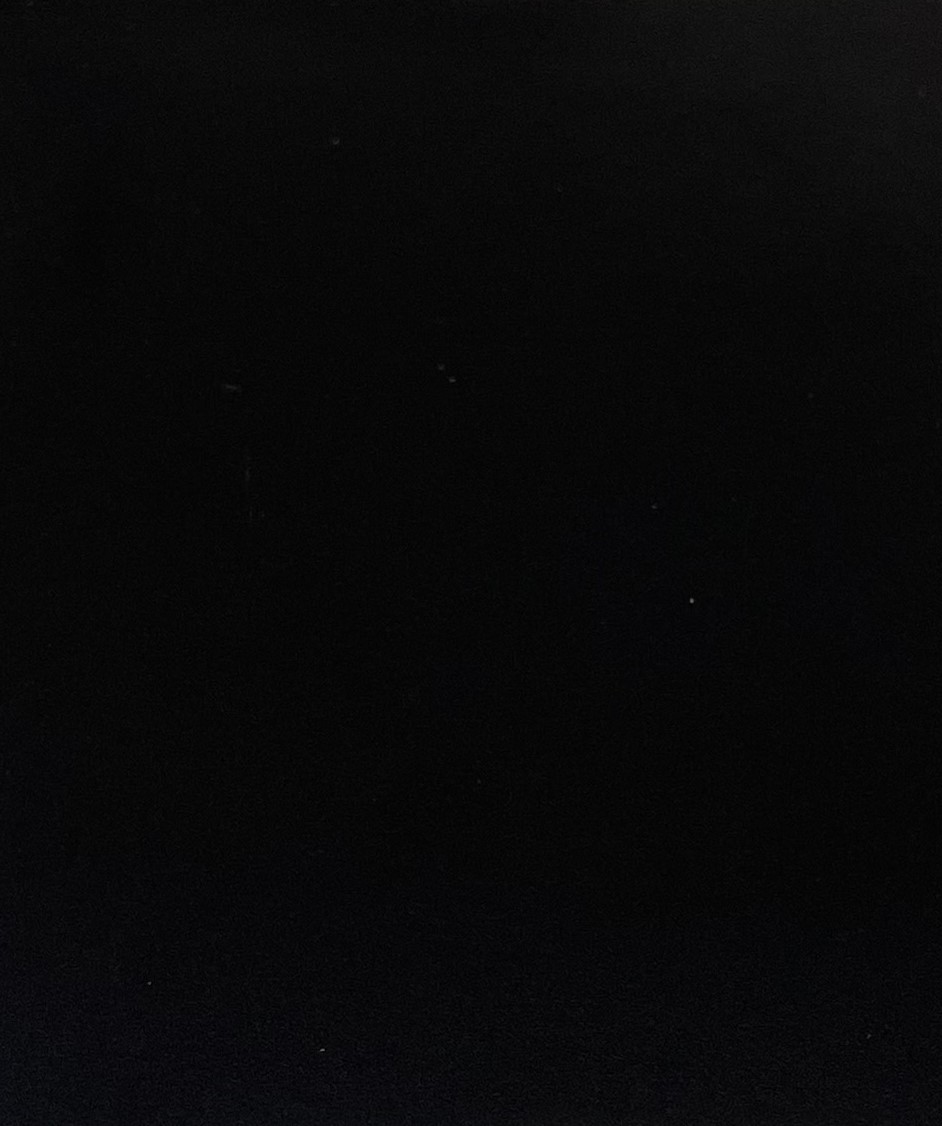

Supplement: S2 File — (ZIP) [file pone.0311343.s002.zip › S2 File/Black (7).JPG]

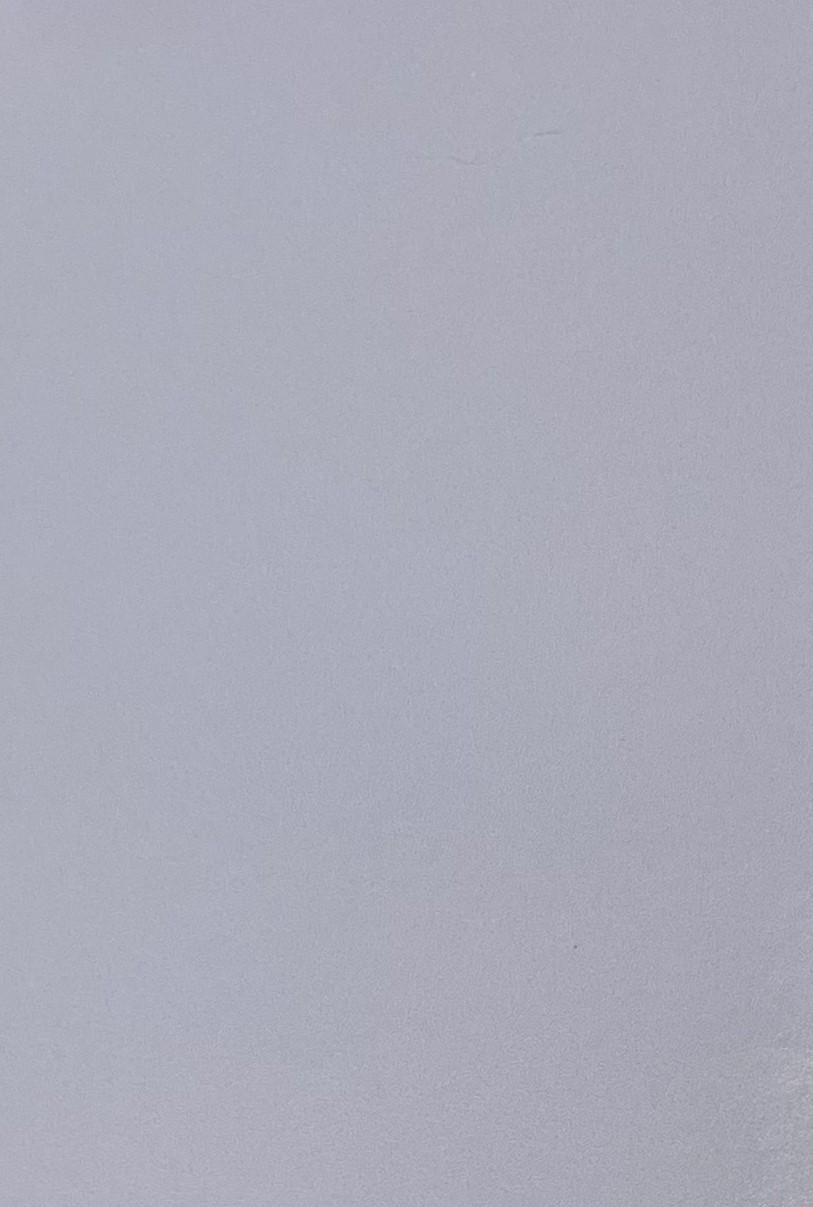

Supplement: S2 File — (ZIP) [file pone.0311343.s002.zip › S2 File/White (7).JPG]

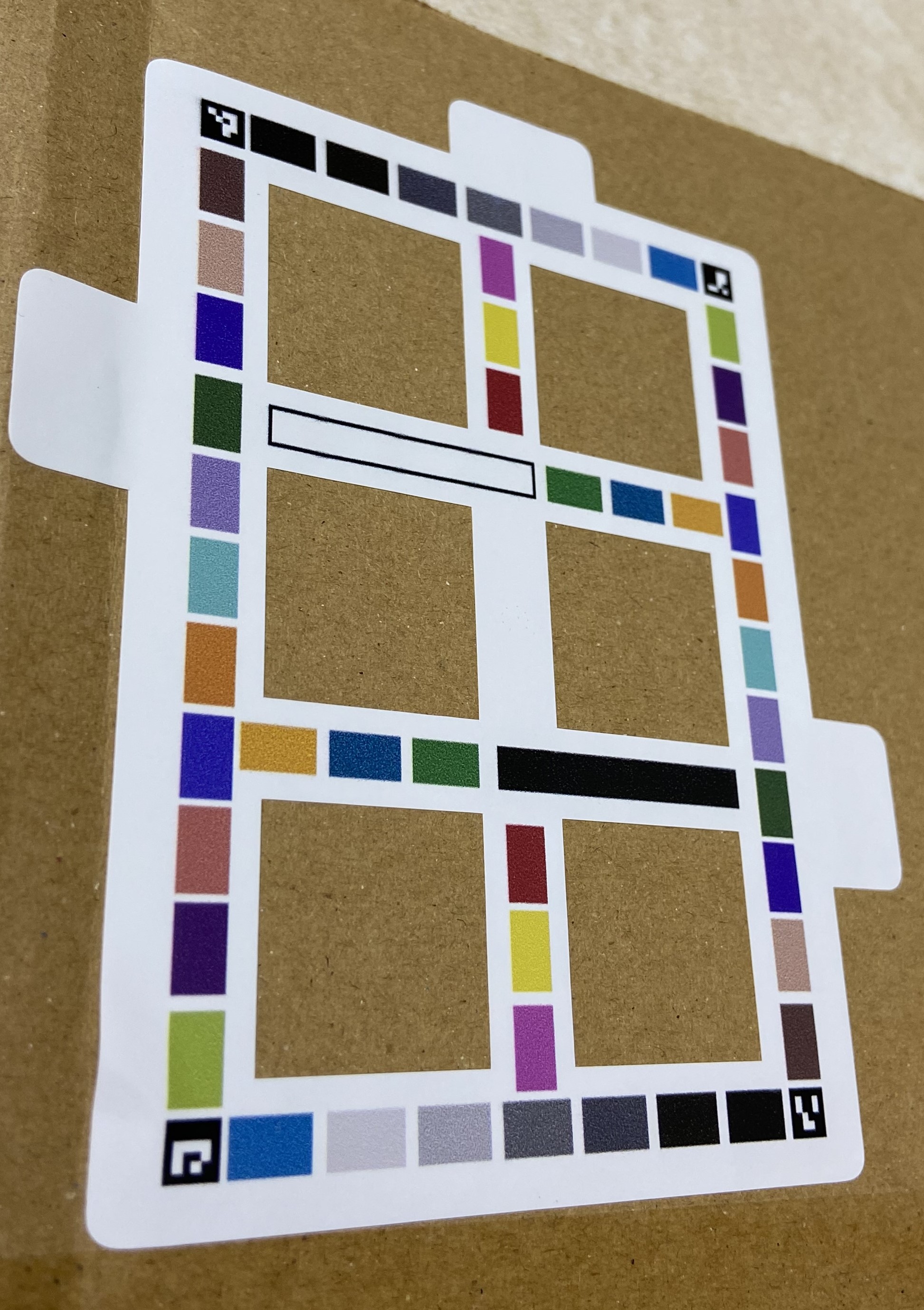

Supplement: S3 File — (ZIP) [file pone.0311343.s003.zip › S3 File/IMG_0039.JPG]

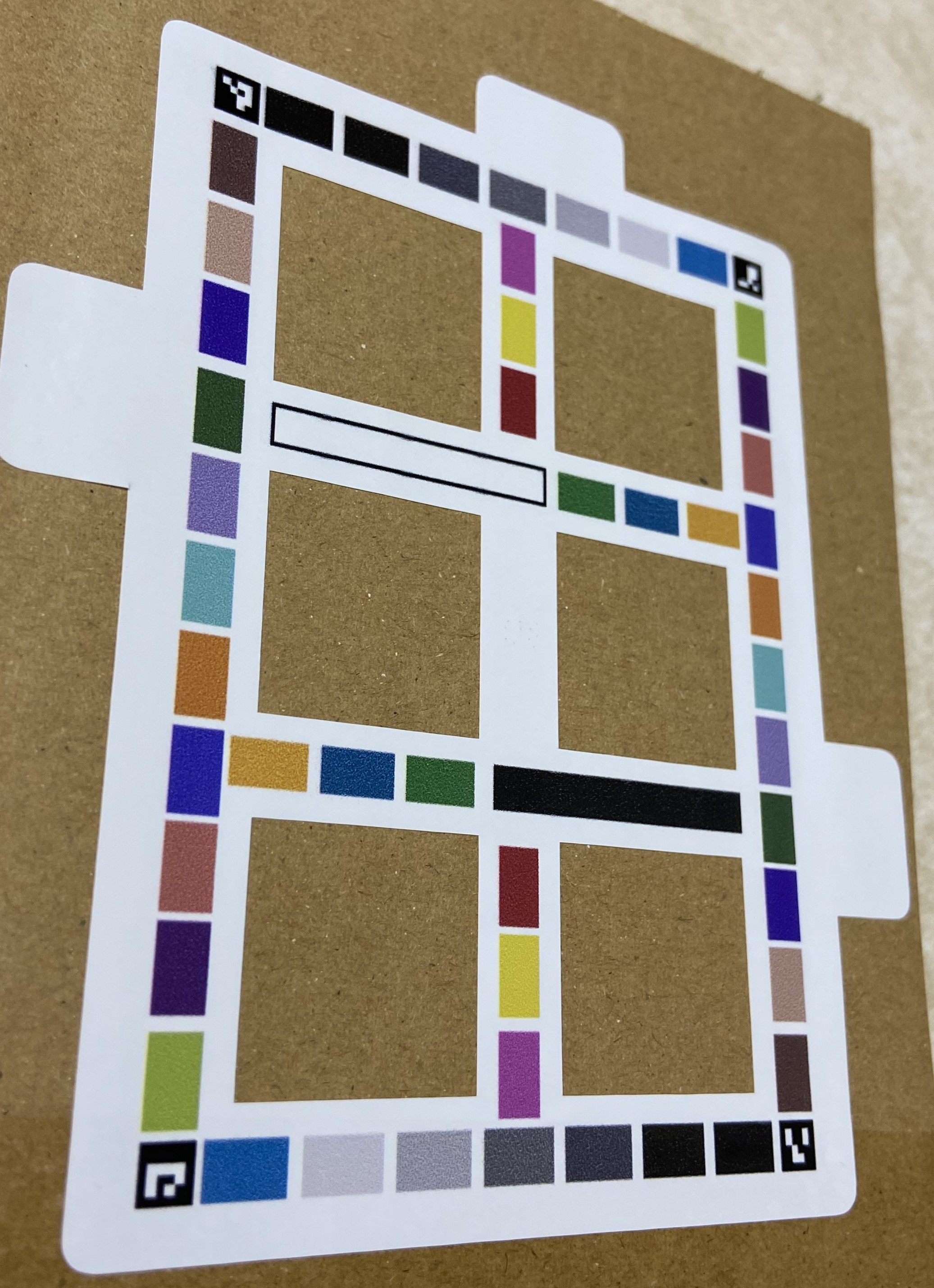

Supplement: S3 File — (ZIP) [file pone.0311343.s003.zip › S3 File/IMG_0038.JPG]

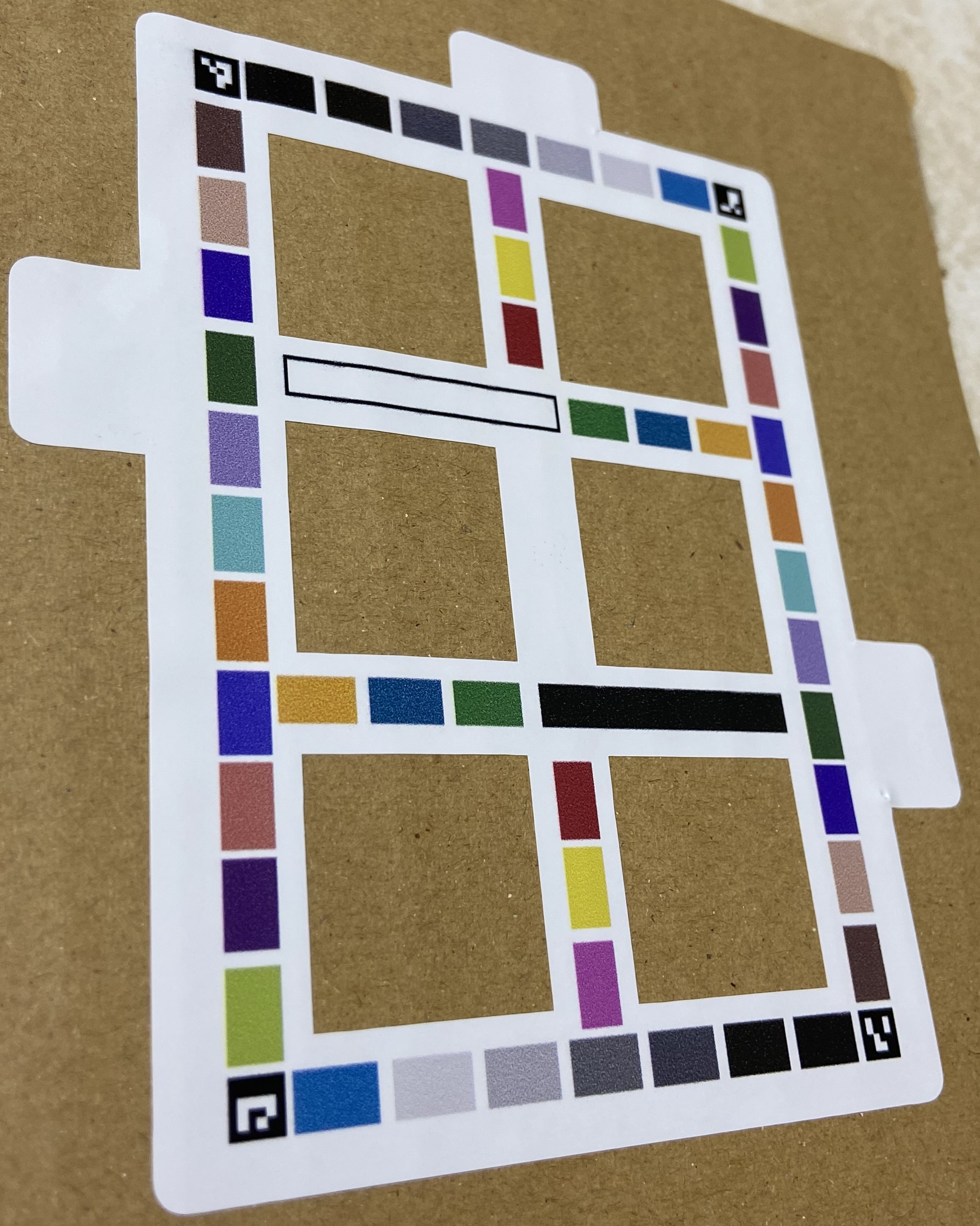

Supplement: S3 File — (ZIP) [file pone.0311343.s003.zip › S3 File/IMG_0042.JPG]

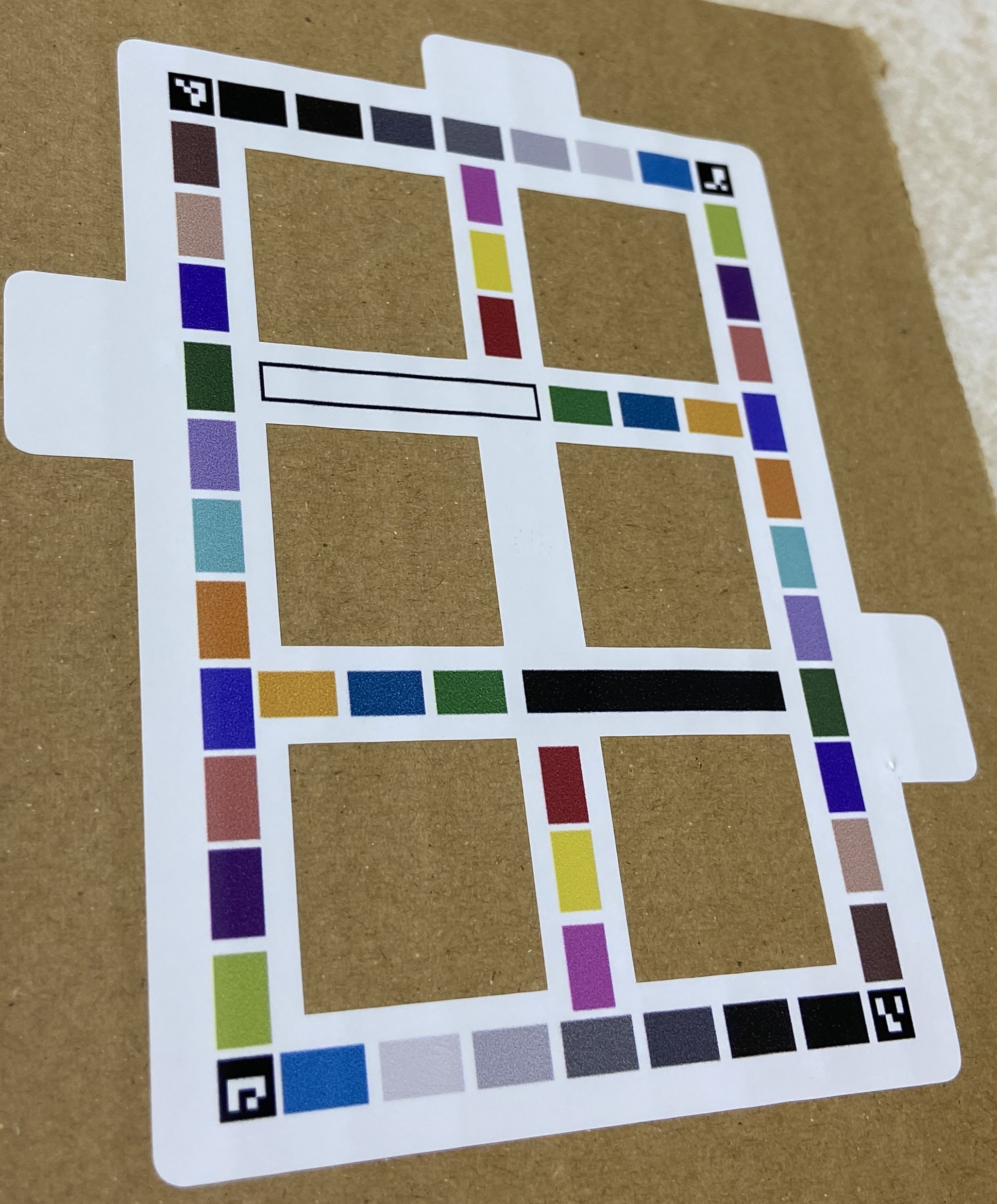

Supplement: S3 File — (ZIP) [file pone.0311343.s003.zip › S3 File/IMG_0043.JPG]

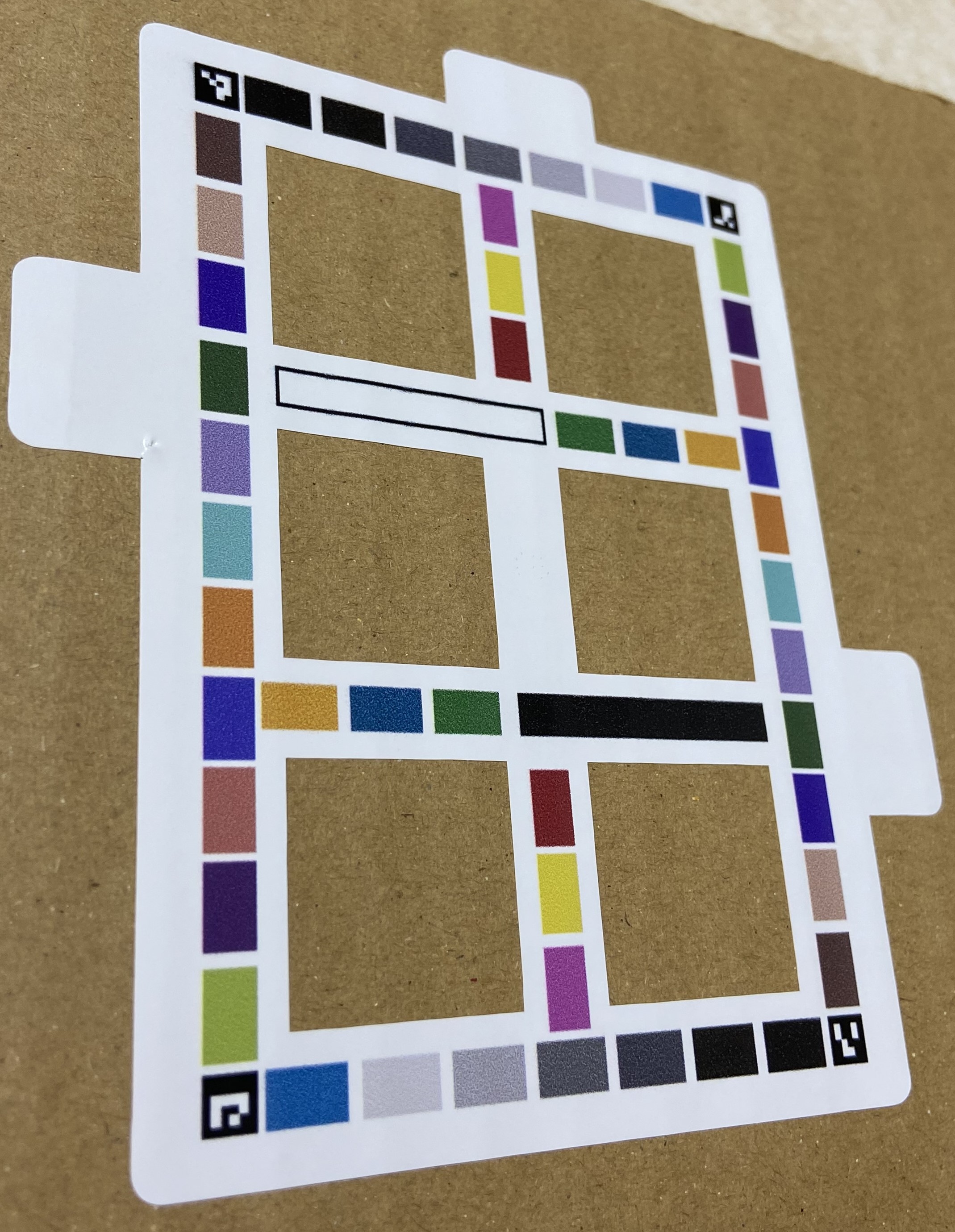

Supplement: S3 File — (ZIP) [file pone.0311343.s003.zip › S3 File/IMG_0041.JPG]

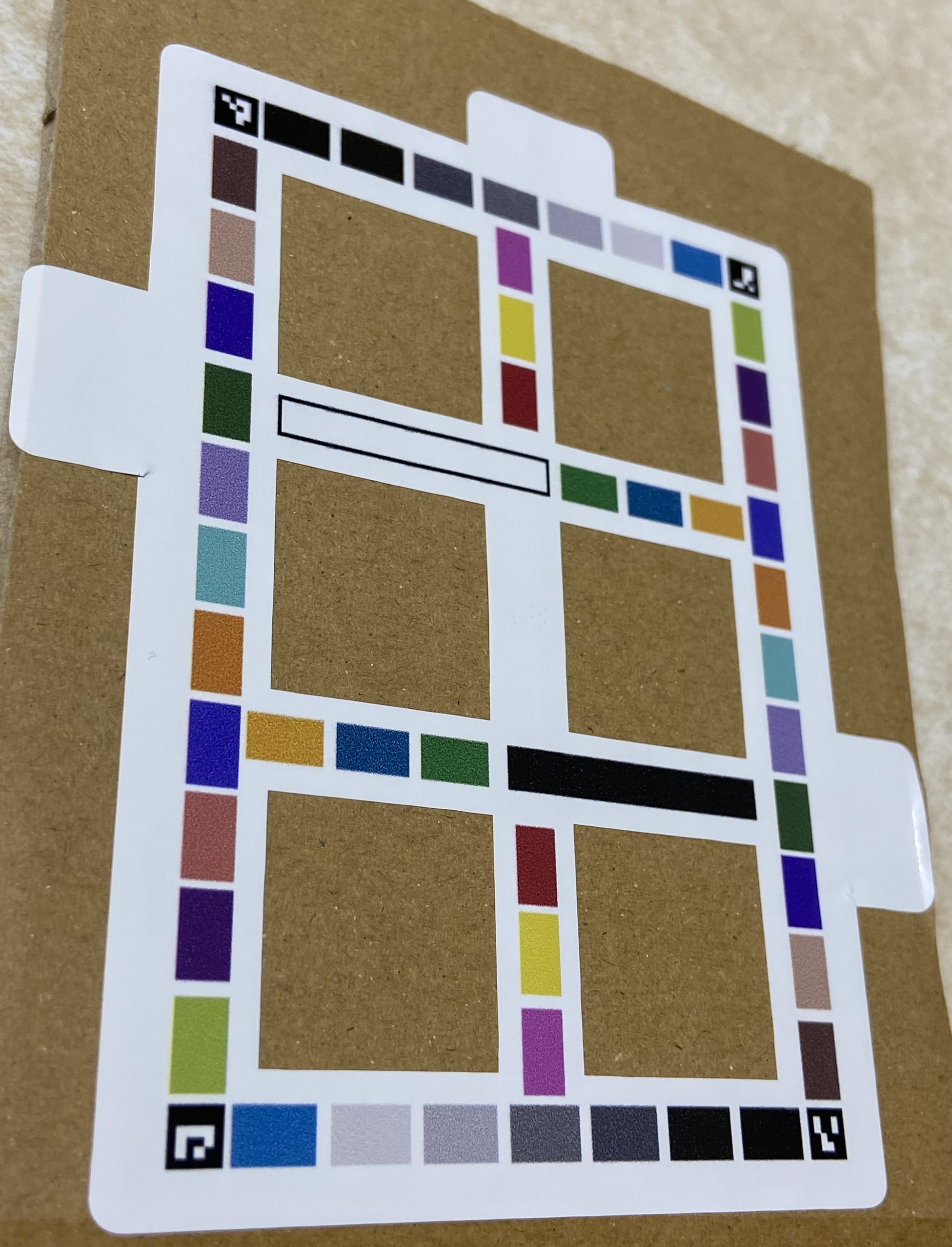

Supplement: S3 File — (ZIP) [file pone.0311343.s003.zip › S3 File/IMG_0040.JPG]

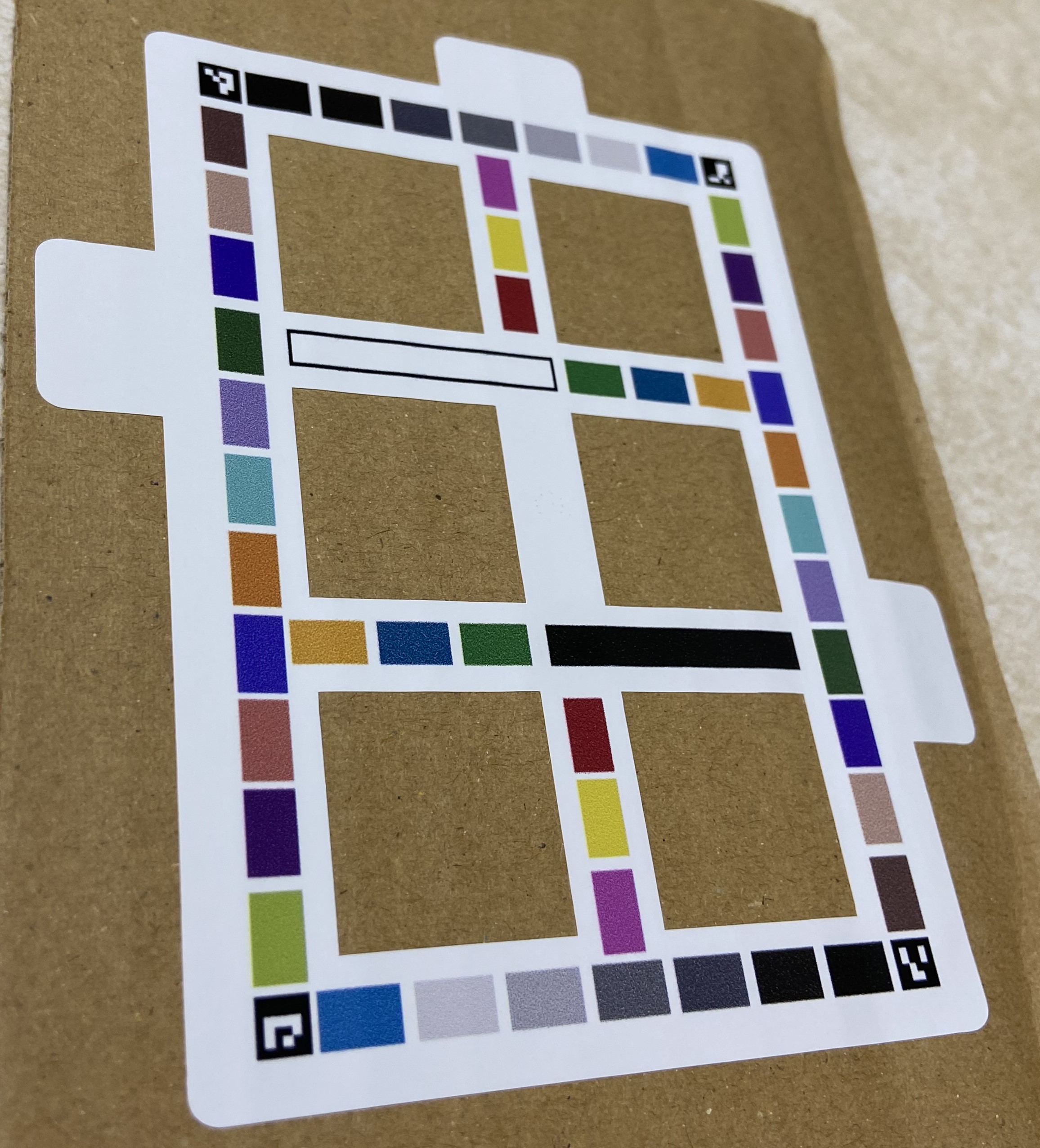

Supplement: S3 File — (ZIP) [file pone.0311343.s003.zip › S3 File/IMG_0044.JPG]

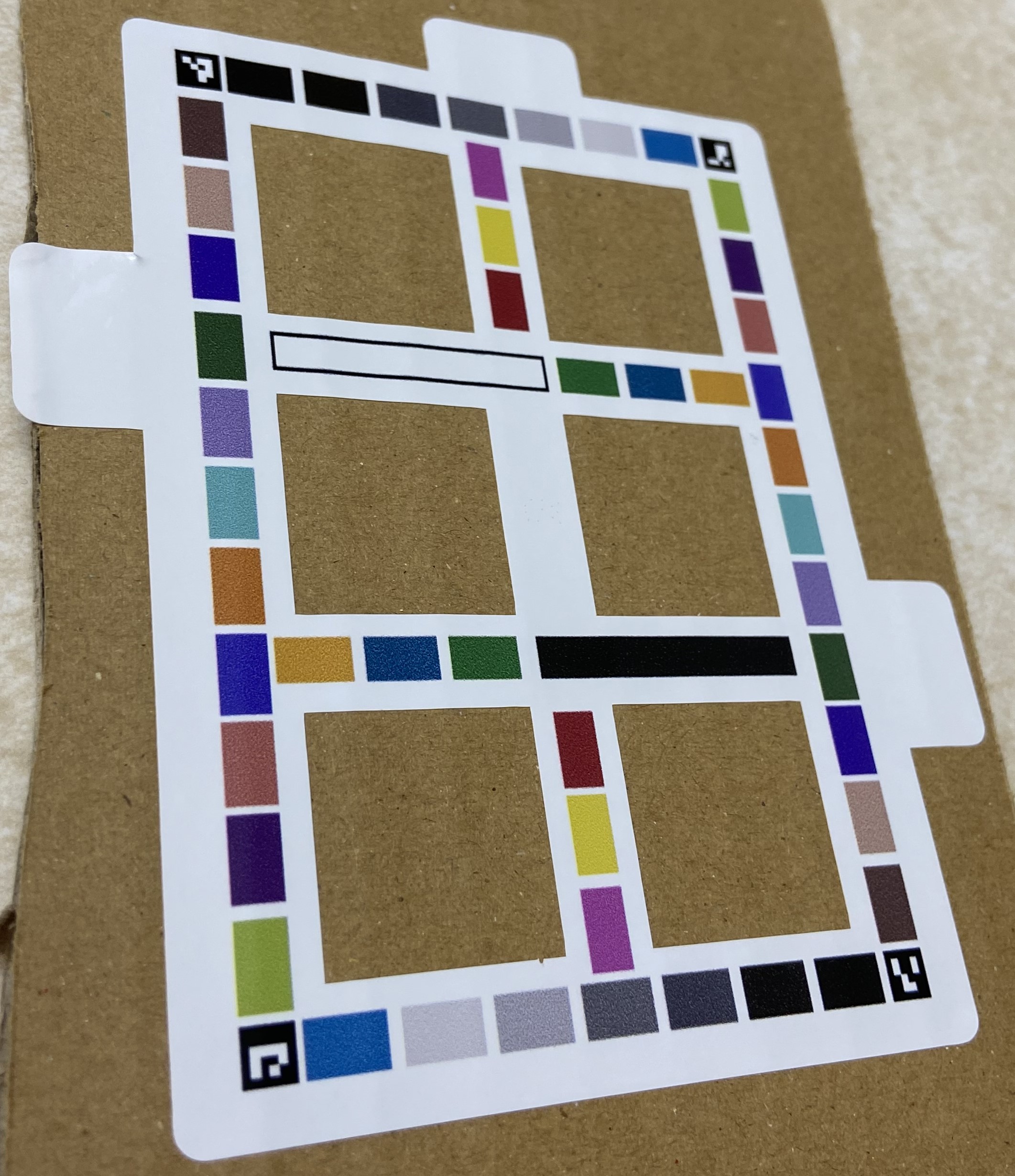

Supplement: S3 File — (ZIP) [file pone.0311343.s003.zip › S3 File/IMG_0045.JPG]

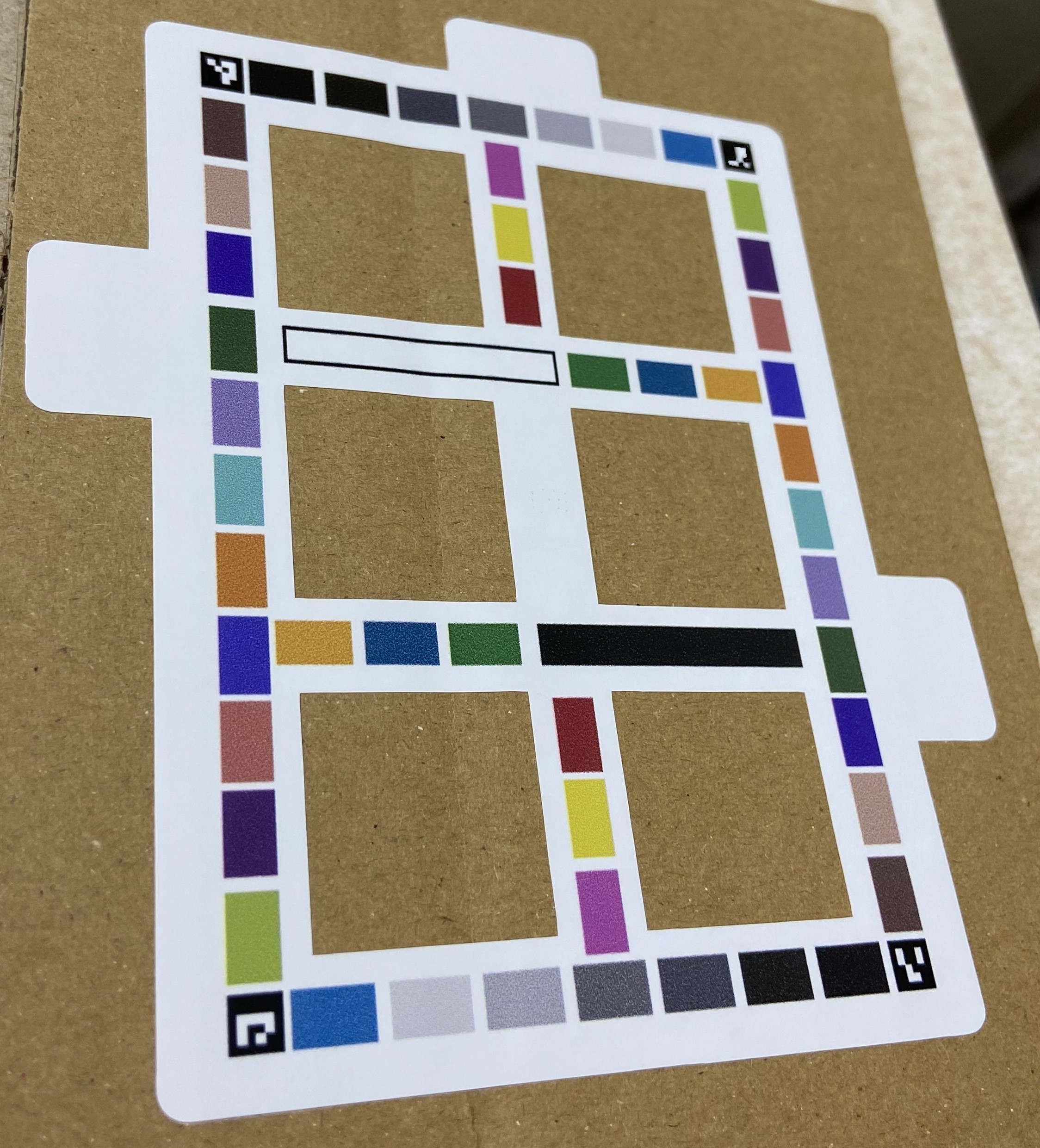

Supplement: S3 File — (ZIP) [file pone.0311343.s003.zip › S3 File/IMG_0046.JPG]

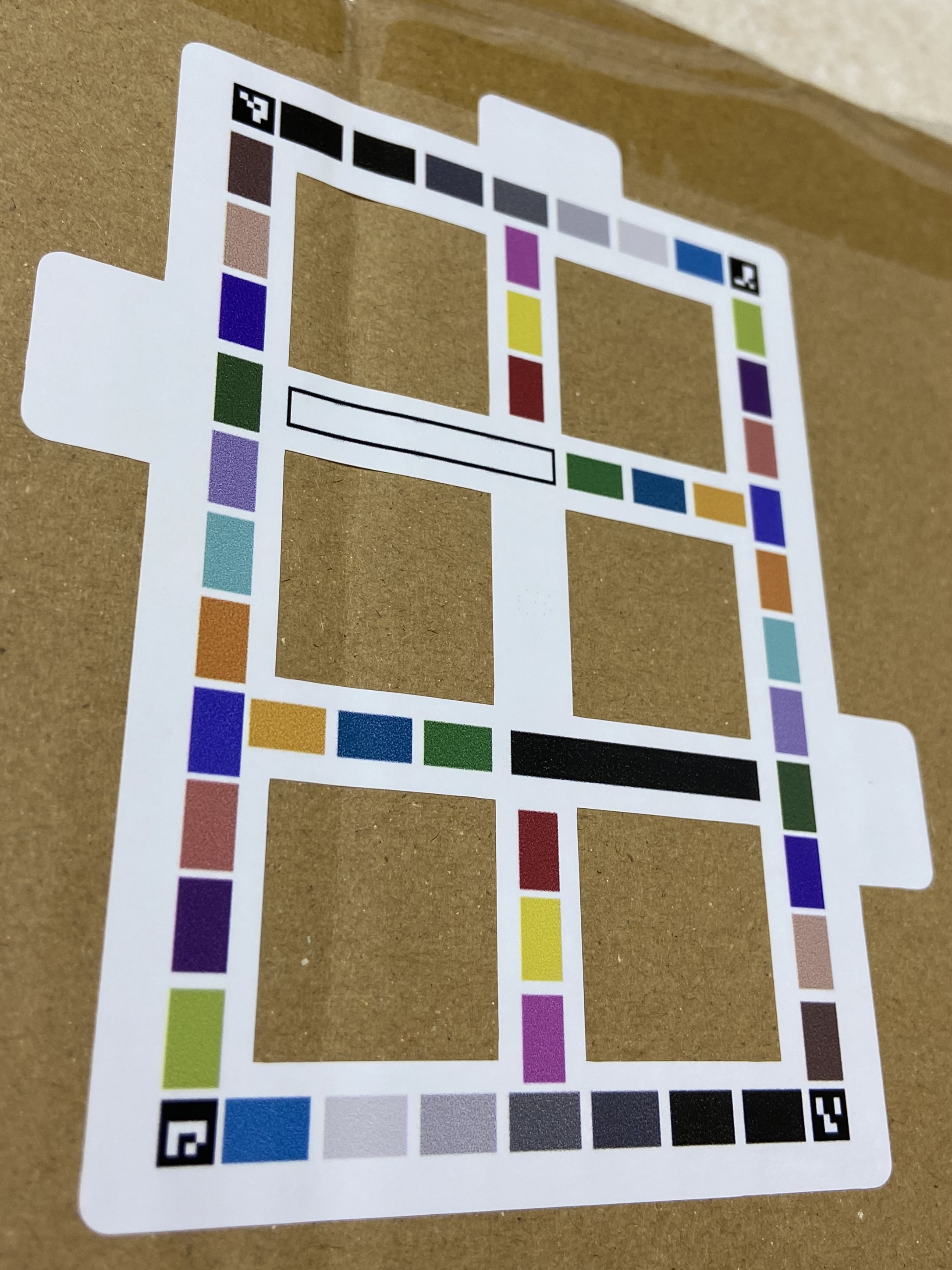

Supplement: S3 File — (ZIP) [file pone.0311343.s003.zip › S3 File/IMG_0037.JPG]
